# Defining Molecular Treatment Targets for Bladder Pain Syndrome/Interstitial Cystitis: Uncovering Adhesion Molecules

**DOI:** 10.3389/fphar.2022.780855

**Published:** 2022-03-25

**Authors:** Guldal Inal-Gultekin, Zeliha Gormez, Naside Mangir

**Affiliations:** ^1^ Department of Physiology, Faculty of Medicine, Istanbul Okan University, Tuzla, Turkey; ^2^ Department of Applied Bioinformatics, Bingen Technical University of Applied Sciences, Bingen am Rhein, Germany; ^3^ Department of Urology, Hacettepe University Hospital, Ankara, Turkey

**Keywords:** gene expression, adhesion molecules, targeted treatment, rare urinary disease, bioinformatics

## Abstract

Bladder pain syndrome/interstitial cystitis (BPS/IC) is a debilitating pain syndrome of unknown etiology that predominantly affects females. Clinically, BPS/IC presents in a wide spectrum where all patients report severe bladder pain together with one or more urinary tract symptoms. On bladder examination, some have normal-appearing bladders on cystoscopy, whereas others may have severely inflamed bladder walls with easily bleeding areas (glomerulations) and ulcerations (Hunner’s lesion). Thus, the reported prevalence of BPS/IC is also highly variable, between 0.06% and 30%. Nevertheless, it is rightly defined as a rare disease (ORPHA:37202). The aetiopathogenesis of BPS/IC remains largely unknown. Current treatment is mainly symptomatic and palliative, which certainly adds to the suffering of patients. BPS/IC is known to have a genetic component. However, the genes responsible are not defined yet. In addition to traditional genetic approaches, novel research methodologies involving bioinformatics are evaluated to elucidate the genetic basis of BPS/IC. This article aims to review the current evidence on the genetic basis of BPS/IC to determine the most promising targets for possible novel treatments.

## Introduction

Bladder pain syndrome/interstitial cystitis (BPS/IC) is a debilitating pain syndrome with unknown etiology (Ueda et al., 2021). BPS/IC is defined as a rare disease with an ORPHA code of 37202. Although different terminologies have been used in the literature, BPS/IC refers to “a clinical syndrome characterized by the complaint of suprapubic pain related to bladder filling, accompanied by other symptoms, such as increased daytime and nighttime frequency, in the absence of proven urinary infection or other obvious pathology” (van de Merwe et al., 2008). As the definition implies, BPS/IC is essentially a pain syndrome defined by excluding other causes and pain mainly perceived as related to the bladder with co-existing lower urinary tract symptoms (such as urgency and frequency). There are no disease biomarkers that can aid in diagnosis (Neuhaus et al., 2021). BPS/IC remains largely unknown in many aspects, including its epidemiology, pathophysiology, and clinical characteristics (Akiyama et al., 2020), which has direct implications on its treatment which is still symptomatic with limited efficiency that inevitably adds to the suffering of patients.

The exclusion of confusable diseases is the mainstay in the diagnosis of BPS/IC. Cystoscopy is usually necessary to rule out underlying pathologies and to screen for typical bladder lesions associated with BPS/IC patients (Parsons et al., 2021). Although cystoscopic examination may be completely normal in a significant proportion of patients, reduced bladder capacity, glomerulations on cystodistension, and Hunner’s lesion (Hanno et al., 2015) can be diagnostic for BPS/IC. Therefore, BPS/IC presents in a large spectrum in the clinic. However, several phenotyping systems have been suggested, which are mainly based on cystoscopic findings, such as Hunner’s lesion BPS/IC and non-Hunner’s lesion BPS/IC. This may be useful in the evaluation, management, and follow-up of patients, as well as in basic scientific research.

The genetic basis of BPS/IC is not fully elucidated. BPS/IC is known to have an association with other unknown pain syndromes such as fibromyalgia and irritable bowel syndrome. Furthermore, BPS/IC was more prevalent in first-degree relatives of women with BPS/IC and monozygotic twins (Cassão et al., 2019). Nevertheless, genes related to the immune system and pro-inflammatory chemokines have been investigated in one study (Ogawa et al., 2010), and a few others tried to define differentially expressed genes (DEGs). However, a specific molecular pathway and potential targets for treatment have not been identified yet.

Like other rare diseases, real advancements in the treatment of BPS/IC necessitate the use of novel, non-traditional research methodologies when designing basic scientific and clinical studies (Kesselheim and Gagne, 2014). Moreover, collaborations between countries and joint work of researchers and clinicians from different disciplines are encouraged to make the best use of the limited patient data that is available. A key area of collaborative work in BPS/IC research is in the area of molecular biology. Transcriptome data can be used as a common source of large-scale molecular data. High-throughput methods, such as microarray technology, create vast quantities of genomic and expression data, which are readily accessible through numerous electronic databases. This massive amount of data can be analyzed using various methods and statistical approaches. One of the most frequent first strategies before wet lab investigations is bioinformatics, which is the intersection point of biology, information, and computational sciences (Hanauer et al., 2007). The computational method combines several databases, including text-mining, and employs multiple statistical analyses on the expression microarray data, thus allowing for the emergence of a more comprehensive overview of the pathology.

A bioinformatic approach was previously adopted by a few other researchers (Gamper et al., 2009; Liu et al., 2020; Saha et al., 2020) to study gene expression profiles. However, the utility of comparing data on different clinical phenotypes has not been investigated before. The current study aimed to compare DEGs in different disease phenotypes, including Hunner’s lesion and non-Hunner’s lesion BPS/IC using various bioinformatic analytical tools and publicly available transcriptome data, with the end purpose of identifying viable targets for BPS/IC therapy.

## Methods

### Interstitial Cystitis Gene Expression Data Sources

An initial search was conducted on NCBI GEO datasets using the search term BPS/IC, and only gene expression arrays in humans were selected for further analysis. Animal studies and the studies that compared BPS/IC with other diseases were excluded. Three different datasets were downloaded from the gene expression omnibus (GEO) database (GENEONTOLOGY, 2021), including GSE11783 (Illumina) (Gamper et al., 2009), GSE28242 (Illumina) (Blalock et al., 2012), and GSE57560 (Illumina) (Colaco et al., 2014).

### Identification of Differentially Expressed Genes

An online easy-to-use interactive web tool, GEO2R (Barrett et al., 2013), was used to analyze the raw data of microarrays and identify differentially expressed genes (DEGs) between patient and healthy control groups. GEO2R uses moderated t-statistic to compare gene expression levels in different groups. The *p*-value < 0.05 and logarithmic fold change |log2FC| ≥ 2 were used as the threshold to obtain statistically significant DEGs. Hence, upregulated genes (*p*-value < 0.05, log2FC ≥ 2) and downregulated genes (*p*-value < 0.05, log2FC ≤ −2) were grouped depending on their expression levels in respect to the cut-off values. The cut-off values were kept at a stringent level to identify the most prominent probes.

### Experimental Design

The grouping within the datasets was kept unmodified as described in each corresponding dataset: GSE11783, GSE28242, and GSE57560. This allowed for the pooling of the probesets from the three datasets in respect to their pathology in two subgroups and one control group. The first subgroup corresponded to BPS/IC patients with Hunner’s lesion and/or other features of more advanced disease such as low bladder capacity (HLD). The second group was composed of BPS/IC patients without Hunner’s lesion and/or with normal bladder capacity (non-HLD). Normal healthy samples from each dataset were pooled together (Control, Ctrl). Patient numbers in each dataset, general information on GEO datasets, and platforms are provided in Supplementary Table S1. Patient and control samples of each dataset are listed in Supplementary Table S2. To warrant homogeneity of samples, male subjects have been excluded from the analysis when possible.

Comparisons were made between the three subgroups: HLD, non-HLD, and Ctrl. The analysis was restricted to the common upregulated and downregulated probesets. Up- and downregulated probes were visualized with volcano plots using the *bioinfokit* tool (Bedre, 2021). Common DEGs for up- and downregulated probesets in the three datasets (GSE11783, GSE28242, GSE57560) were identified with Venn diagrams using the online tool “Bioinformatics and Evolutionary Genomics” (Bioinformatics and Evolutionary Genomics, 2021). Heat maps and hierarchical clustering analysis were performed with the *bioinfokit* tool (Bedre, 2020). To create the expression matrix used to generate a heat map of common genes and samples, the most significant probe (the smallest *p*-value) was selected across all probes representing a gene.

### Protein–Protein Interaction Construct, Functional Enrichment, and Pathway Analysis

Protein–protein interaction (PPI) networks for differentially expressed genes (DEGs) were built. Gene Ontology (GO) enrichment and pathway analysis of up- and downregulated probesets were performed using the Enrichr Classification System database (Chen et al., 2013; Kuleshov et al., 2016; Xie et al., 2021). Enrichr is an easy-to-use gene set enrichment analysis tool. The genes were enriched for biological process (BP), molecular functions (MF), and cellular components (CC). The Enrichr database provides for a comparison of a variety of pathway databases for a single inquiry. For this study, the Kyoto Encyclopedia of Genes and Genomes (KEGG) pathway analysis was selected, as it was one of the most recently updated databases (KEGG 2021) (Kanehisa et al., 2021).

### Candidate Hub Protein Identification

Candidate hub genes were identified using the cytoHubba in the Cytoscape v3.8.2 software. cytoHubba is a ready-to-use plug-in that predicts and explores significant nodes and sub-networks. All genes were sorted by degree score, and hub genes were restricted to the top ten.

## Results

The up- and downregulated probesets for each dataset were visualized with Volcano plots (Supplementary Figure S1). Among the statistically up-and downregulated probesets of the three datasets (GSE11783, GSE28242, and GSE57560), the comparison with Venn diagrams for the upregulated probesets between three comparison groups—HLD vs. non-HLD, HLP vs. Ctrl, and non-HLD vs. Ctrl—revealed 116, 185, and 7 overlapping probesets, respectively (Supplementary Figure S2, Supplementary Table S3). Similarly, the comparison for the downregulated probesets revealed 30, 122, and 1 overlapping probesets, respectively (Supplementary Figure S2, Supplementary Table S4).

A specific clustering of genes was not detected in the three dataset heat maps analysis; however, a clustering was noticeable in the downregulated genes for the HLD vs. Ctrl comparison (Supplementary Figure S3).

### Protein–Protein Interaction Construct and Gene Ontology Enrichment Analysis

**FIGURE 1:**
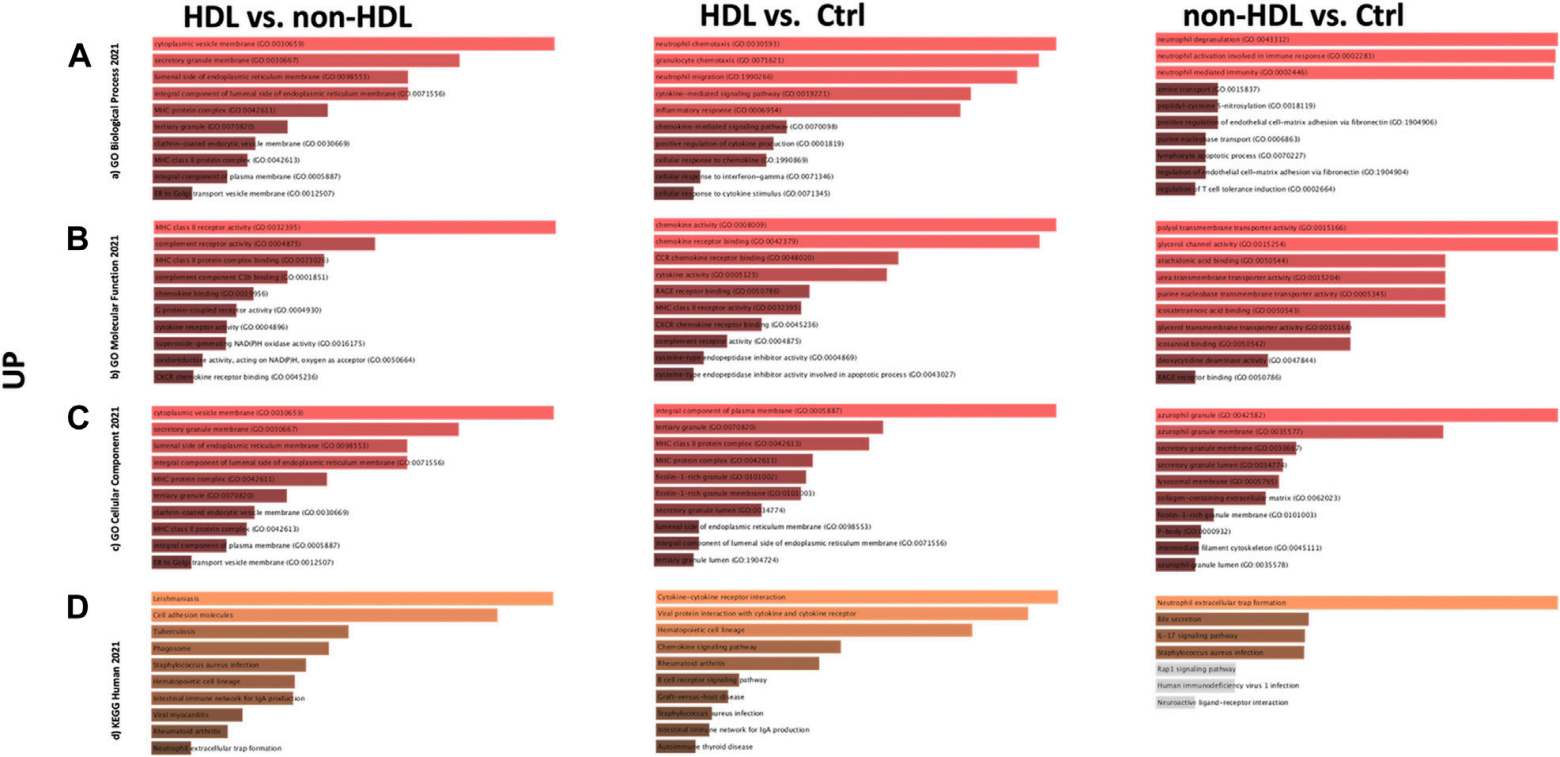
Gene Ontology (GO) and putative pathways of common upregulated DEGs in BPS/IC obtained for HLD vs. non-HLD, HLD vs. Ctrl, and non-HLD vs. Ctrl subgroup analysis. The GO terms were extracted from the Enrichr platform in the form of bar graphs for each subgroup analysis, in which the color and length of the bars decrease as the significance decreases. Significant (*p*-value < 0.05) GO terms were analyzed for **(A)** biological processes, **(B)** molecular functions, and **(C)** cellular components aspects, and **(D)** top 10 significant putative pathways predicted with the Enrichr platform obtained from the Kyoto Encyclopedia of Genes and Genomes (KEGG).

**FIGURE 2:**
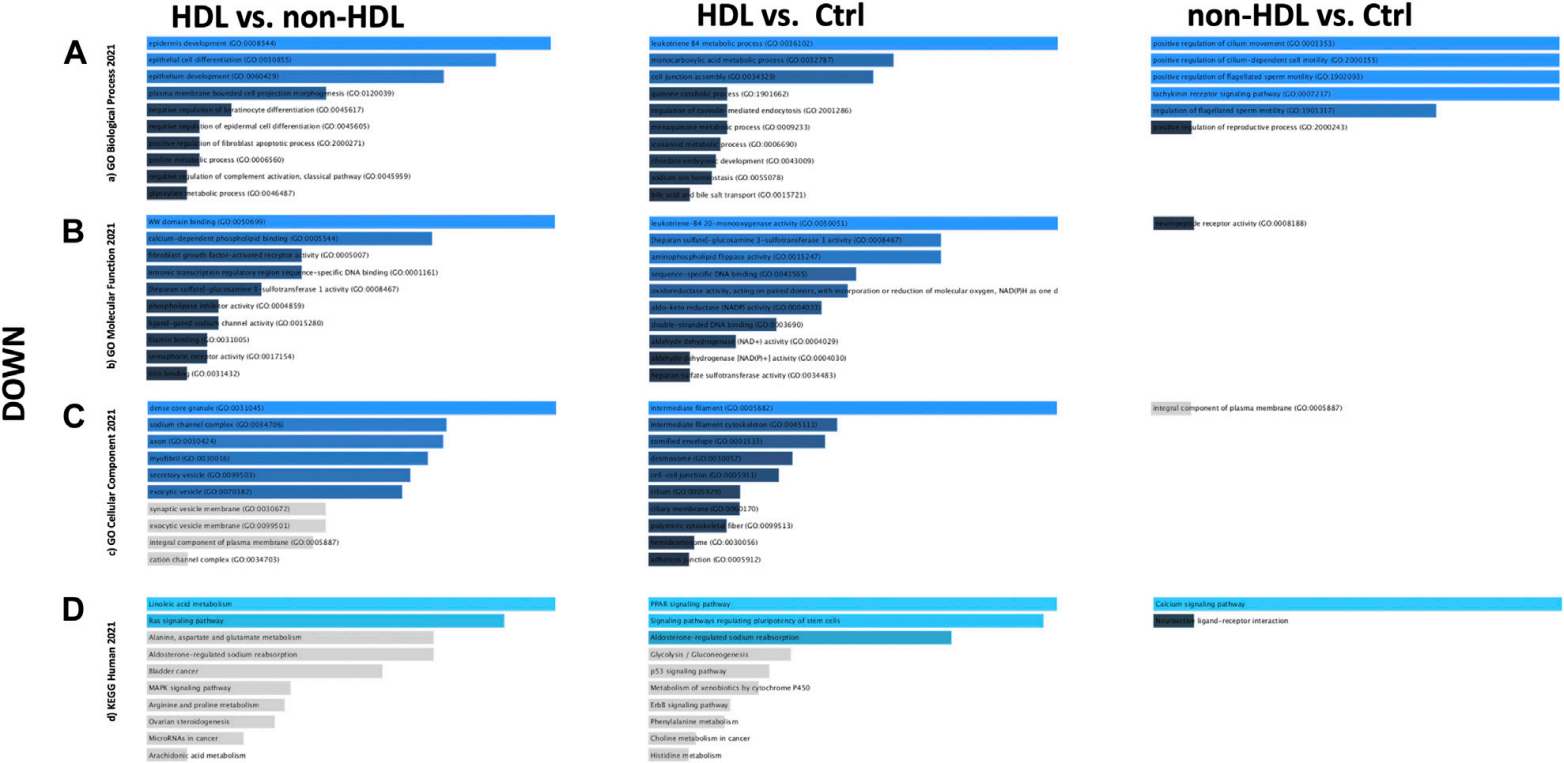
Gene Ontology (GO) and putative pathways of common downregulated DEGs in BPS/IC obtained for HLD vs. non-HLD, HLD vs. Ctrl, and non-HLD vs. Ctrl subgroup analysis. The GO terms were extracted from the Enrichr platform in the form of bar graphs for each subgroup analysis, in which the color and length of the bars decrease as the significance decreases. Significant (*p*-value < 0.05) GO terms were analyzed for **(A)** biological processes, **(B)** molecular functions, and **(C)** cellular components aspects, and **(D)** top 10 significant putative pathways predicted with the Enrichr platform obtained from the Kyoto Encyclopedia of Genes and Genomes (KEGG).

PPIs were built for the three-group comparisons using the common up- and downregulated probes of the three datasets. The probes were enriched for the BP, MF, and CC aspects for all three comparison groups. Among the three datasets, a Gene Ontology (GO) enrichment analysis was performed for the mutually significant 116, 185, and 7 upregulated (Figures 1A–C) and 30, 122, and 1 downregulated (Figures 2A–C) probesets with Enrichr, for each comparison group (HLD vs. non-HLD, HLD vs. Ctrl, non-HLD vs. Ctrl). The genes were enriched for BP, MF, and CC aspects and provided the top ten significant terms in a graphical format which was also available in a tabular format. Among the upregulated probesets for each analysis group (HLD vs. non-HLD, HLD vs. Ctrl, and non-HLD vs. Ctrl), the top 10 occupants for BP, MF, and CC were terms related to immunity. For HLD vs. non-HLD analysis, GO ontologies revealed significance for cytoplasmic vesicle membrane, secretory granule membrane, and MCH class II protein complex terms for BP, MHC class II activity and complement receptor activity for MF, and endoplasmic reticulum membrane and MCH class II protein activity for CC. For HLD vs. Ctrl, analysis of the GO enrichment demonstrated neutrophil and granulocyte chemotaxis, migration for BP, chemokine and MHC class II receptor activity for MF, and MHC class II protein complex for CC. The non-HLD vs. Ctrl comparison GO analysis showed significant enrichment for “neutrophil degranulation” for BP and “azurophil granules” for CC. As for the downregulated probesets, “epithelial cells differentiation” GO term was among the significant terms for BP (HLD vs. non-HLD), “cell junction assembly” for BP, and “desmosome” and “cell-cell junction” for CC (HLD vs. Ctrl).

### Pathway Analysis

The pathways observed from KEGG for the commonly upregulated probesets for each comparison group are revealed as follows: cell adhesion molecules pathway was the second most significant pathway within the 116 mutual proteins for HLD vs. non-HLD; cytokine-cytokine receptor interaction was the most significant pathway and cell adhesion molecules pathway was the 12th most significant predicted pathway for the 185 overlapping proteins for HLD vs. Ctrl; and among the seven common proteins for non-HLD vs. Ctrl, only seven pathways were predicted, where the “neutrophil extracellular trap (NET) formation” was first in line (Figure 1D; Table 1). Among the commonly downregulated probesets, “calcium signaling pathway” and the “neuroactive ligand-receptor interaction” pathways for non-HLD vs. Ctrl comparison were predicted to be significant (Figure 2D).

**TABLE 1:** Pathway analysis for commonly upregulated probes in each analysis group and their respective *p*-values, odds ratio, and predicted enriched proteins within each pathway (HLD: Hunner’s lesion disease; non-HLD, non- Hunner’s lesion disease; Ctrl, control).

	Term	*p*-value	Odds ratio	Genes
HLD vs. non-HLD	Cell adhesion molecules (2nd row)	1.484E-13	20.431062509236	CD274; SELPLG; ITGA4; **ITGB2;** HLA-B; ITGAL; **PTPRC**; HLA-DMB; SELL; HLA-DPB1; HLA-DRA; CD226; HLA-DQA2; HLA-DQB1
	Neutrophil extracellular trap formation (10th row)	1.330E-18	11.59796682718031	CR1; SELPLG; NCF1; PRKCB; CLEC7A; **ITGB2**; FPR1; CYBB; TLR8; FPR3; ITGAL
HLD vs. Ctrl	Cytokine-cytokine receptor interaction (1st row)	4.922E-15	10.200821532316631	CCL23; CCL21; TNFRSF9; CXCR5; IL5RA; TNFRSF11B; CXCL1; PPBP; CXCL13; CXCL2; **IL6**; CCL8; IL2RA; IL21R; **CD27**; **CCL2**; **CCR7**; LTB; CCL19; CCL18; TNFRSF4; RELT; CCL17
	Cell adhesion molecules (10th row)	8.999E-7	8.439670697195782	**SELL**; CD6; CD28; HLA-DPB1; **CTLA4**; ICOS; TIGIT; HLA-DOB; HLA-DQA1; HLA-DQB1
non-HLD vs. Ctrl	Neutrophil extracellular trap formation (1st row)	0.001808	42.36577540106952	**AQP9**; **FPR1**

a The genes/proteins in bold are also hub proteins identified with the cytoHubba.

### Hub Protein Identification

**FIGURE 3:**
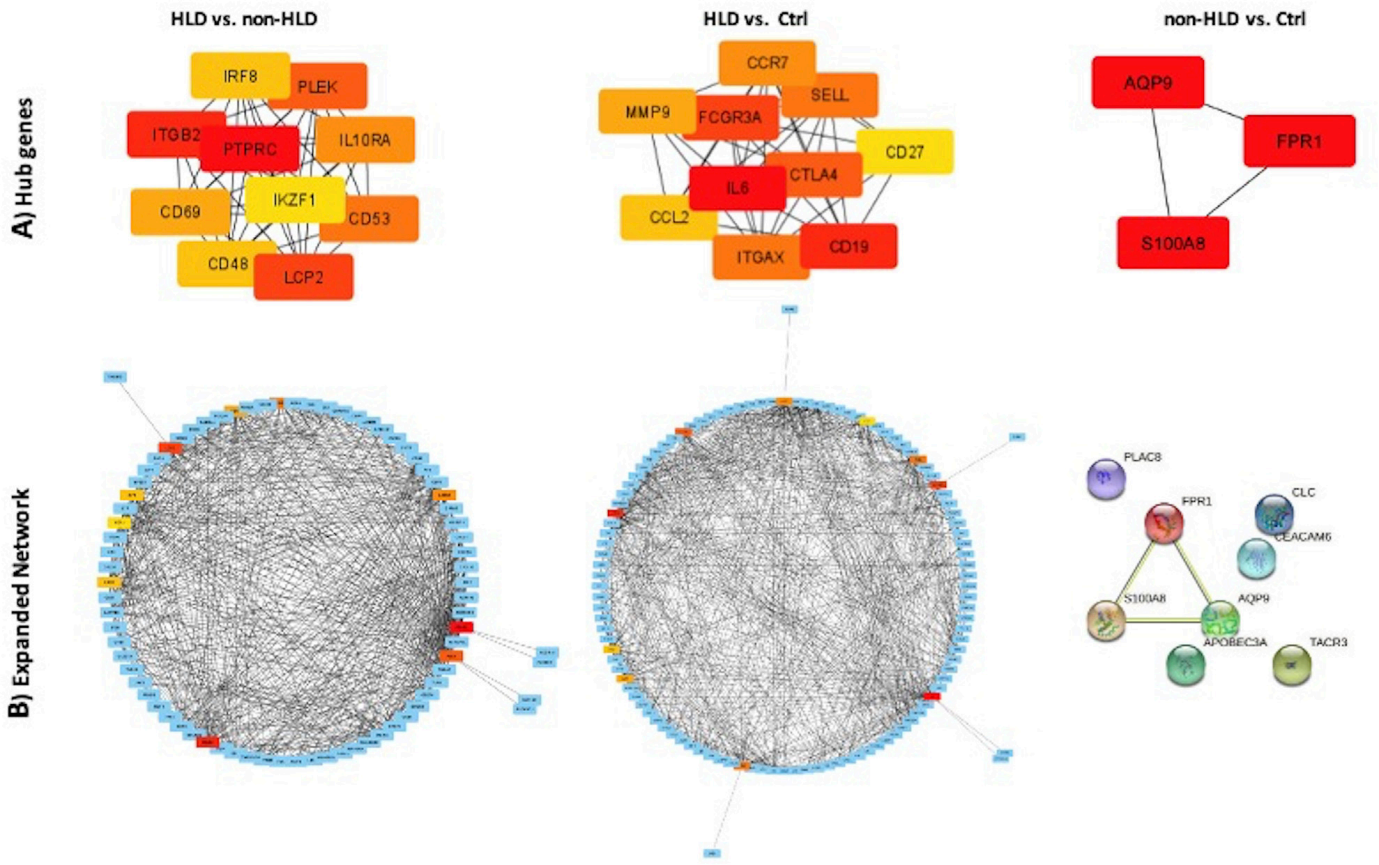
PPI network of common genes and hub genes were identified using Cytoscape and cytoHubba plug-in, respectively, for HLD vs. non-HLD, HLD vs. Ctrl, and non-HLD vs. Ctrl subgroup analysis. **(A)** Hub genes are colored from yellow to red, with red being the most important. **(B)** Expanded network with first-stage nodes of hub genes; from yellow to red color indicating higher importance for red hub genes; and blue nodes represent DEGs.

For the three group comparisons, cytoHubba was used to find hub genes. The identified hub genes showed close relation as represented in the STRING format for three analyses: HLD vs. non-HLD, HLD vs. Ctrl, and non-HLD vs. Ctrl (Figures 3A,B).

## Discussion

This study identified several hub genes/proteins and pathways coding the molecules, expressed on the leukocytes and epithelial cells, which imply an increased inflammation and cell adhesion processes in BPS/IC. Our approach was novel in that we have performed bioinformatic analysis combining and comparing the available datasets in clinically meaningful phenotypes of patient groups. Although this approach considerably increased the number of performed analyses and restricted the number of patients with pathology by keeping them in separate groups (Hunner’s lesion and non- Hunner’s lesion), this has provided a new perspective on the involvement of prevalent molecular expressions and pathways at various phases, including the transition from healthy to low pathology compared to the development of advanced disease.

Comparing pooled data from different phenotypes of BPS/IC, three genes were differentially upregulated in patients with low pathology, *AQP9, S100A8*, and *FPR1.* These genes are responsible for promoting cell migration, chemotaxis, and regulation of cell volume of neutrophils and monocytes. Therefore, they mainly act in the early phases of inflammation. Further comparisons between patients with Hunner’s lesion revealed other differently upregulated genes, including but not limited to *ITGB2*, *ITGAX*, *CD53*, *CD69*, *SELL, IL-6*, *CTLA4*, *CCL2*, and *CHI3L1.* These genes are responsible for the maintenance of inflammation and therefore are thought to play a role in chronic inflammation.

### Enriched GO Terms for Pathology Comparisons

The GO terms for non-HLD vs. Ctrl comparison predict increased “neutrophile activity and degranulation” for the GO: MF aspect accompanied with an increased “activity in azurophil granules” for the GO: CC aspect. GO terms for HLD vs. non-HLD comparison predict “local inflammation”; demonstrating increased “antigen presentations with MHC antigen receptor and complex”, whereas the HLD vs. Ctrl comparison demonstrates a full-blown inflammatory response with increased “chemotaxis and migration of polymorphonuclear cells” and “increased cytokine and chemokine signaling,” still accompanied with “MHC class II receptor activity.”

In terms of GO: MF, the different ranks of antigen processing molecules (1st for HLD vs. non-HLD; 6th for HLD vs. Ctrl) are noteworthy. This demonstrates the physiological escalation of an immune response, which begins with chemotaxis and migration and then shifts to antigen presentation and signaling, which is complemented with adhesion molecules.

### Predicted Prevailing Pathways for Pathology Comparisons

The NET formation was the most significant pathway for non-HLD vs. Ctrl comparison for the upregulated common DEGs. Moreover, the Ca signaling pathway was significantly predicted for downregulated DEGs. Cell adhesion pathways prevailed for the HLD vs. non-HLD (2nd row) comparison, and the NET pathway was significantly predicted (10th row). Several inflammatory and immune pathways dominated the top 10 predicted pathways for the HLD vs. Ctrl comparison, while the cell adhesion pathway occupied the 12th row. When examining the pathology’s progression, the anticipated pathways make sense, allowing one to observe a growing inflammatory response step by step, as indicated by the three groups in this study.

The observed NET formation in the early stages of the disease demonstrates the initiation of the inflammatory processes. NETs are DNA scaffolds surrounded by granule-derived proteins from neutrophils and eosinophils, and they have been described as part of immune system defense mechanisms not just for antimicrobial defense but also for a range of sterile inflammatory and autoimmune diseases (Yousefi et al., 2008; Lande et al., 2011; Boeltz et al., 2019). There is evidence that BPS/IC is an inflammatory condition (Cervigni and Natale, 2014). The predicted NET pathways in this study for non-HLD vs. Ctrl and HLD vs. non-HLD comparisons along with the chemotaxis and adhesion pathways support the presence of a sterile inflammation process that escalade into the debilitating syndrome with its accompanying manifestations.

On the contrary, this analysis has demonstrated the presence of a strong inflammatory response in the advanced pathology group, with several cytokines, and chemokine signals, creating increased chemotaxis and migration of neutrophils.

As for the difference between advanced (HLD) and early (non-HLD) pathology stages, the comparison reveals an increase in the expression of antigen-presenting receptors, complement binding regions, and chemokine receptors, thus the activity in the intracellular membranes (BP, MF, and CC aspects) in the HLD group, which collectively produce enhanced cell adhesion pathways. Simultaneously, there is still a considerable persistence of NET formation, implying that the inflammatory process will continue.

Apart from the scrutinized pathways herein, rheumatoid arthritis and autoimmune thyroid disease pathways were also predicted to be significantly related to the common upregulated DEGs. This suggests that these DEGs can be associated with autoimmune diseases. In addition to the hub genes when investigated, some have been previously reported for Graves disease, Hashimoto thyroiditis, celiac disease, and systemic lupus erythematosus.

Furthermore, the heat maps did not generate clear clusters of genes/proteins for the three comparison groups. This might be due to low numbers of overlapping genes/proteins for disease groups (HLD and non-HLD). This could be partly attributable to the clinical heterogeneity of patients with advanced pathology included in this study.

### Hub Genes and Associated Diseases

The predicted hub genes for the analyses support the significant pathways and GO enrichment terms. Only three hub genes were identified for non-HLD vs. Ctrl comparison, *AQP9*, *S100A8*, and *FPR1*. *AQP9* was previously reported in the top 25 hub gene list in the Gamper and colleagues (2009) in their gene expression profile study conducted with ulcerative IC (Gamper et al., 2009). AQP9 is a member of a subset of aquaporins called aquaglyceroporins and encodes a protein that is reported to play an active role in the volume regulation of neutrophils and their migration (Karlsson et al., 2011). *FPR1* encodes a receptor of mammalian phagocytic cells and mediates their response to invasion by activating microbicidal, secretory, and chemotactic functions *in vitro* (Murphy et al., 1993). *S100A8* is a member of the S100 superfamily of proteins containing calcium-binding regions. The protein calprotectin comprises S100A8 and S100A9 subunits, which are abundantly expressed on neutrophils, monocytes, and early differentiation stages of macrophages. When S100A8/S100A9 complex is released by activated granulocytes, the complex acts as a cytokine and bind to cell surface receptors, which trigger signaling pathways involved in the inflammatory processes. The complex plays critical roles in numerous cellular processes, including cell cycle progression, cell survival, proliferation, differentiation, and cell migration (Koy et al., 2013; Shabani et al., 2018).

Hub genes in the HLD vs. non-HLD comparison are all observed to be part of immune system signaling pathways, with most of them being surface molecules. Some of the 10 hub genes were examined in further depth.

*PTPRC*, also known as CD45, is a major naïve leukocyte cell surface molecule. It is essential for activation of T and B cells by mediating cell-to-cell contacts and is also involved in integrin-mediated adhesion and migration of immune cells (Jacobsen et al., 2000).

*CD53* encodes a cell surface panleukocyte glycoprotein that is known to complex with integrins, and contributes to the transduction of CD2-generated signals by T cells and natural killer cells. The deficiency of this protein is linked to immunodeficiency with recurrent infectious diseases (Angelisová et al., 1990). The prominence of naïve lymphocytes in the HLD group in comparison to the non-HLD group suggests an initiation in the pathology.

*ITGB2* (CD18) encodes the beta subunit common to the three alpha integrin chains *ITGAL* (CD11A), *ITGAM* (CD11B), and *ITGAX* (CD11C). These cell surface membrane glycoproteins form leukocyte-specific integrins. Their function is to promote adherence of neutrophils and monocytes to stimulated endothelial cells. ITGB2 protein genetic defects in *ITGB2* are associated with leukocyte adhesion deficiency (Barclay et al., 1993). *CD48* is a member of the CD2 subfamily of immunoglobulin-like receptors and a surface protein of lymphocytes and endothelial cells (Yokoyama et al., 1991). *CD69* encodes a member of the calcium-dependent lectin surface glycoprotein, which appears at the earliest on lymphoid cells upon activation. It is involved in lymphocyte proliferation and functions as a signal-transmitting receptor in lymphocytes (Cambiaggi et al., 1992). In addition, *CHI3L1* was the single common upregulated gene for all three datasets, encoding the lectin-type YKL-40 cell adhesion protein, one of the main human articular chondrocyte proteins. It is also expressed on activated macrophages and neutrophils (Liu et al., 2020). Although this gene did not appear in the top predicted pathways and hub genes, its expression has previously been reported in the serum and urine samples of IC patients (Richter et al., 2010) and increased expression on macrophages and mast cells in the detrusor muscle (Liu et al., 2020).

The hub genes suggest that, from the early stages of the disease to more severe pathology, the inflammatory process is maintained by increased expression of cell adhesion molecules, including integrins and lectins, which enhance cell-to-cell contact between T and B lymphocytes and possibly other subtypes of leukocytes with epithelial cells of the bladder.

Hub genes for HLD vs. Ctrl comparison display two cell surface adhesion molecules in the top ten list, *ITGAX* integrin and *SELL* a lectin, belonging to a family of adhesion/homing receptors. SELL protein operates with a calcium-binding epidermal growth factor-like domain. It is required for binding and subsequent rolling of leucocytes on endothelial cells, facilitating their migration into sites of inflammation (Siegelman and Weissman, 1989). Together with ITGAX, they provide for leukocyte and epithelial cell adhesion during the inflammatory process and other cell adhesion molecules that were not highlighted in the top ten. This comparison, however, yielded more B- and T-cell activators, cytokines, and their receptors, which seems logical as the analyses compared severe/advanced pathology with healthy subjects. Some of the top ten hub genes discussed here characterize a progressive chronic inflammatory process. It is noteworthy that some of the hub genes have been previously associated with autoimmune disorders. *CD19* is a well-known cell surface protein restricted to naïve B lymphocytes. *FCGR3A* encodes a receptor for the Fc portion of immunoglobulin G and is expressed on the natural killer cell surface as a membrane glycoprotein. *IL-6* is a pro-inflammatory cytokine that functions in the maturation of B-cell and T-cell regulation and takes part in the acute phase of inflammation. Additionally, it is acknowledged as an endogenous pyrogen capable of inducing fever in people with autoimmune diseases or infections (Ishihara and Hirano, 2002; Mucida et al., 2007). *CTLA4*, a member of the immunoglobulin family, encodes a protein that transmits an inhibitory signal to T cells. Mutations on this gene have been associated with several autoimmune conditions (Schneider et al., 2006). *CCL2*, also known as monocyte chemotactic protein-1 (MCP1), is one of the several cytokines involved in immunoregulatory and inflammatory processes, generating chemotactic activity for monocytes and basophils (Corrigall et al., 2001). This molecule could be reasonable for BPS/IC as mast cell infiltration is well-documented (Peeker et al., 2000).

Interestingly, a member of the matrix metalloproteinase (MMP) family, *MMP9*, was observed in the top ten hub genes in HLD vs. Ctrl comparison. MMPs are involved in remodeling the extracellular matrix (ECM) in health and disease. MMPs degrade components of ECM during inflammation. Therefore, MMPs have been suggested to play a role in chronic inflammation and tissue fibrosis (Cai et al., 2008). Thus, MMPs might be involved in ECM changes leading to reduced bladder capacity in BPS/IC.

### Possible Treatment Options

Estrogen-related pathways are observed with a higher prevalence in women (Berry et al., 2011). Thus, the very few male samples have not been considered to avoid confusing findings. However, proteins and pathways related to estrogen and progesterone have not been acquired in this study; rather, the findings focused on inflammation and adhesion molecules, bringing to light the concept of anti-adhesion molecular therapy possibilities.

A mechanistic discussion on cell adhesion molecules and their functions and regulations for the immune system exceeds the aim of the study and has been elaborated in detail elsewhere (Albelda et al., 1994; Zundler et al., 2017; Harjunpää et al., 2019). However, the results obtained herein suggest that BPS/IC might benefit from anti-adhesion agents as a potential repurposing treatment. Successful examples of anti-adhesion molecule therapies exist for inflammatory bowel diseases (IBDs), such as Crohn’s disease (CD) and ulcerative colitis (UC), which are, similar to BPS/IC (Riedl et al., 2013), characterized by chronic inflammation, and associated with considerably reduced quality of life (Lönnfors et al., 2014). Currently, a monoclonal antibody is successfully implemented worldwide in the treatment of CD and UC. The concept behind these small molecule antibodies is to inhibit surface molecules on T cells (integrins) that control their ability to attach to the gut’s endothelial surface. Likewise, although the molecular pathophysiology of BPS/IC is yet to be fully understood, an anti-adhesion strategy is proposed here. Molecular and structural binding of adhesion molecules on bladder epithelial cells can be studied, which can pave the road for repurposing treatment strategies.

Up-to-date, three groups of animal models are being used to study BPS/IC: bladder-centric models, models with complex mechanisms, and psychological and physical stressors/natural disease models. Because of the complexity of the clinical presentation, it is recommended that various models be used to disclose the molecular components of BPS/IC (Birder and Andersson, 2018). The hub proteins identified in this study are strong candidates for future studies with animal models.

## Conclusion

In conclusion, we have demonstrated bioinformatically for the first time the genes that were differentially expressed in patients with two different phenotypes of BPS/IC and controls. The genes coding for proteins acting in acute inflammation were active in the early phases of the disease, whereas molecular pathways active in chronic inflammation were more prominent in the later stages of the disease. This suggests that BPS/IC could present in a spectrum regulated by adhesion molecules maintaining acute and chronic inflammation.

The expression levels of the defined molecular targets can be suggested as candidate biomarkers to identify the level of pathology for treatment purposes. Furthermore, the identification of specific anti-adhesion molecules to delay the inflammatory process is proposed. The correct anti-adhesion therapy might assist in reducing the progressive sterile inflammation and benefit the current anti-inflammatory treatment regimens.

## Data Availability

Publicly available datasets were analyzed in this study. This data can be found here: https://www.ncbi.nlm.nih.gov/geo/query/acc.cgi?acc=GSE11783 https://www.ncbi.nlm.nih.gov/geo/query/acc.cgi?acc=GSE28242 https://www.ncbi.nlm.nih.gov/geo/query/acc.cgi?acc=GSE57560.

## Supplementary Material

Supplementary Figure S1 All probesets in three datasets, **(A)** GSE11783, **(B)** GSE28242, and **(B)** GSE57560, visualized by Volcano plots for comparisons between HLD vs. non-HLD, HLD vs. Ctrl, and non-HLD vs. Ctrl subgroup analysis. Each point represents a single probeset, with fold change values on the *x*-axis and logarithmic *t*-test *p*-value on the *y*-axis. Red, blue, and grey dots indicate significantly upregulated (*p* < 0.05, Log2 FC ≧ 2), significantly downregulated (*p* < 0.05, Log2 FC ≦ –2), and insignificant probesets, respectively (HLD, Hunner’ s lesion disease; non-HLD, non- Hunner’ s lesion Disease; Ctrl, control).

Supplementary Figure S2 Common probesets between GSE11783, GSE28242, and GSE57560 datasets in respect to HLD vs. non-HLD, HLD vs. Ctrl, and non-HLD vs. Ctrl subgroup analysis for **(A)** upregulated and **(B)** downregulated probesets were identified using Venn diagrams (HLD, Hunner’ s lesion disease; non-HLD, non- Hunner’ s lesion Disease; Ctrl, control).

Supplementary Figure S3 The heat maps of each dataset for the HLD vs. Ctrl comparison. Each square represents a probeset (*y*-axis) for each sample (*x*-axis). Red and blue colors represent upregulation and downregulation, respectively, whereas the white color represents no change in expression levels. Samples in the black boxes are patients corresponding to the HLD group in each dataset.
