# Supplementary figures and images for: Defining Molecular Treatment Targets for Bladder Pain Syndrome/Interstitial Cystitis: Uncovering Adhesion Molecules
Source: Front Pharmacol. 2022 Mar 25;13:780855. doi: 10.3389/fphar.2022.780855 (PMC8990855; doi:10.3389/fphar.2022.780855)

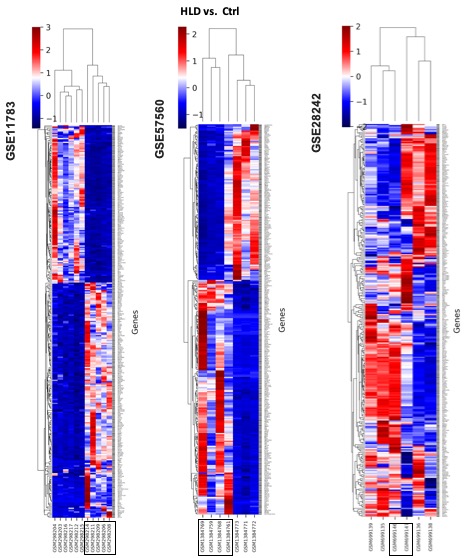

Supplement: Supplementary file 1 [file Image3.JPEG]

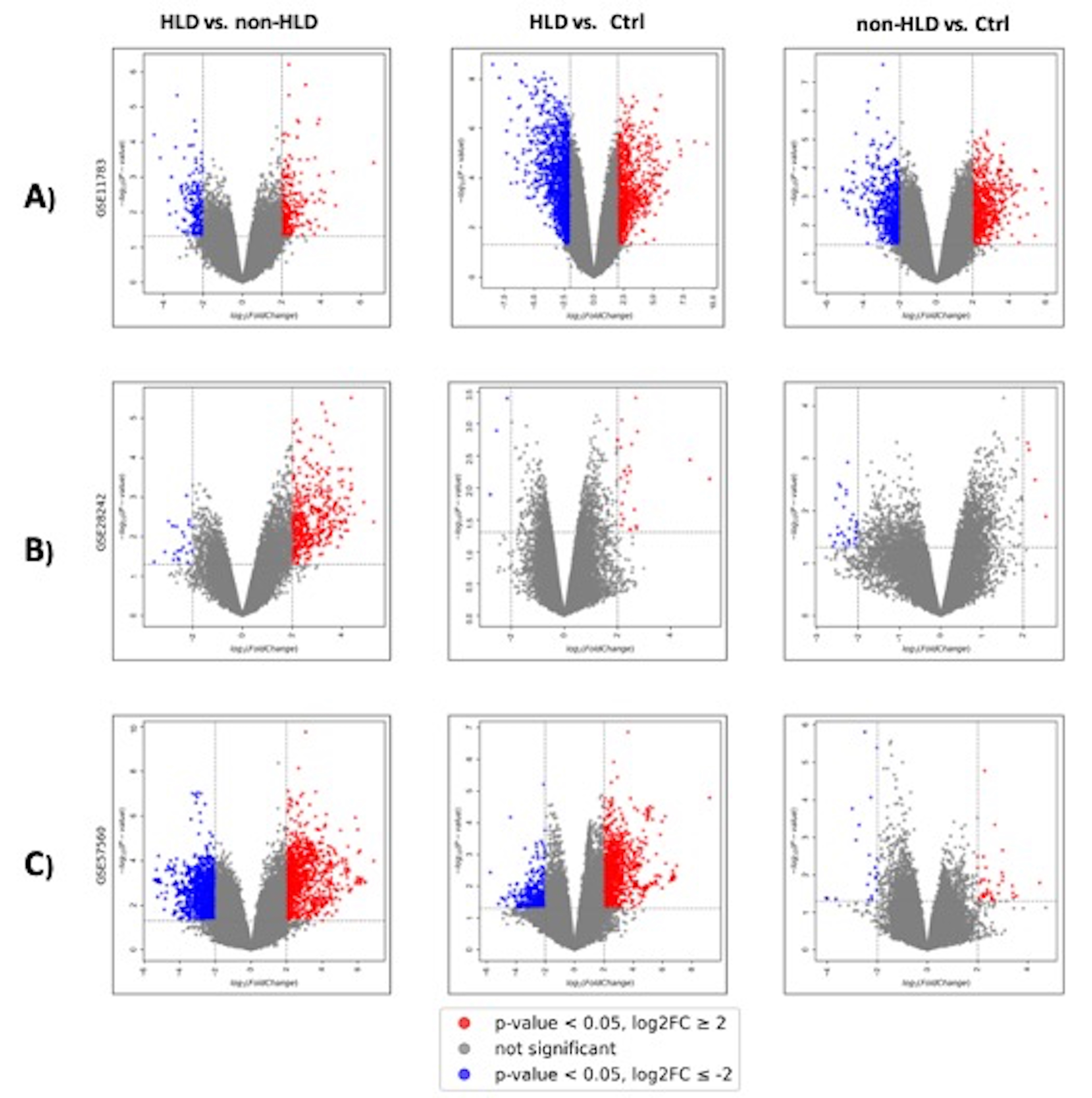

Supplement: Supplementary file 3 [file Image1.JPEG]

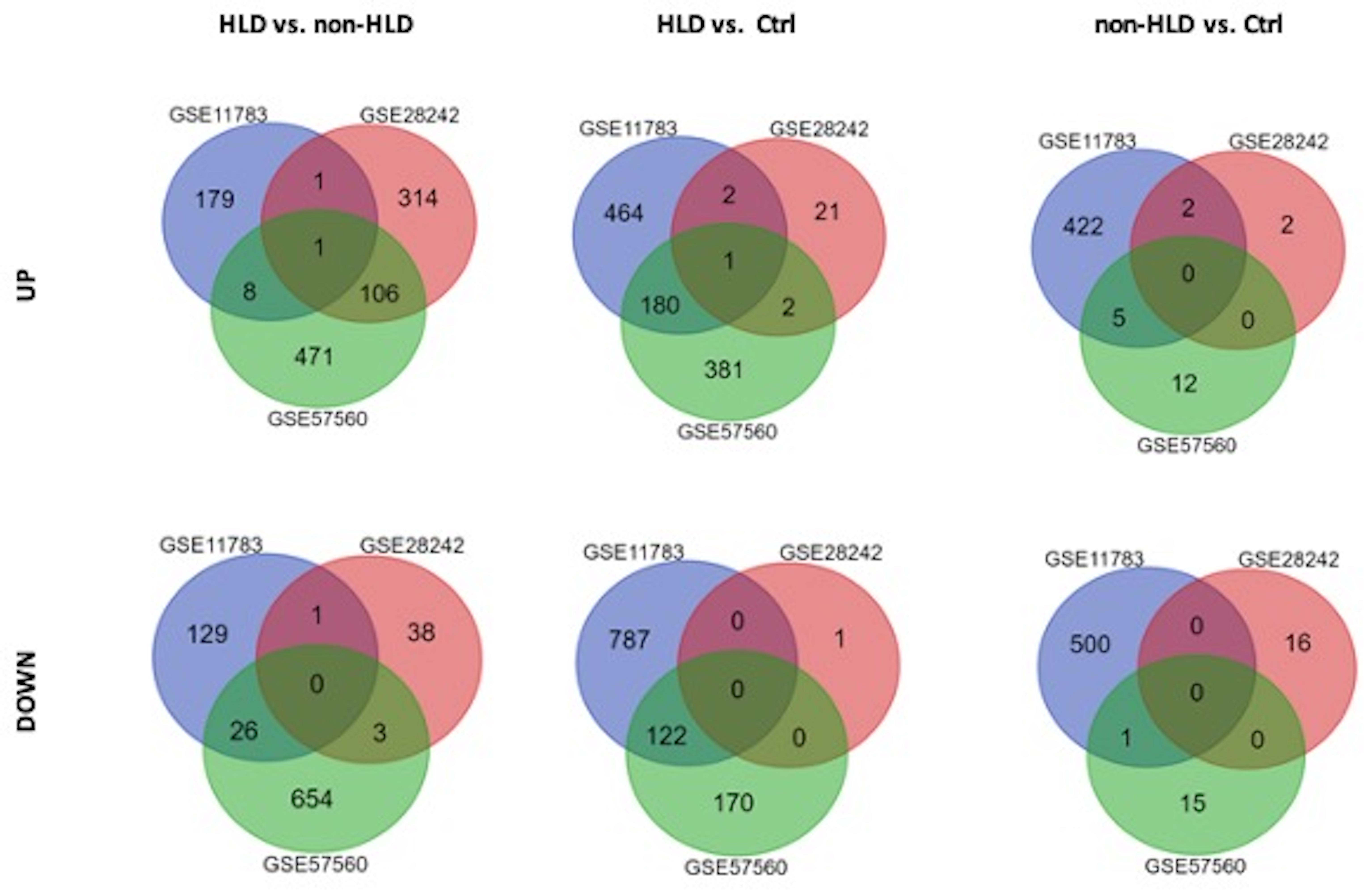

Supplement: Supplementary file 4 [file Image2.JPEG]
