# Supplementary material for: Defining Molecular Treatment Targets for Bladder Pain Syndrome/Interstitial Cystitis: Uncovering Adhesion Molecules
Source: Front Pharmacol. 2022 Mar 25;13:780855. doi: 10.3389/fphar.2022.780855 (PMC8990855; doi:10.3389/fphar.2022.780855)
Supplement: Supplementary file 2 [file Presentation1.pptx]

## Slide 1
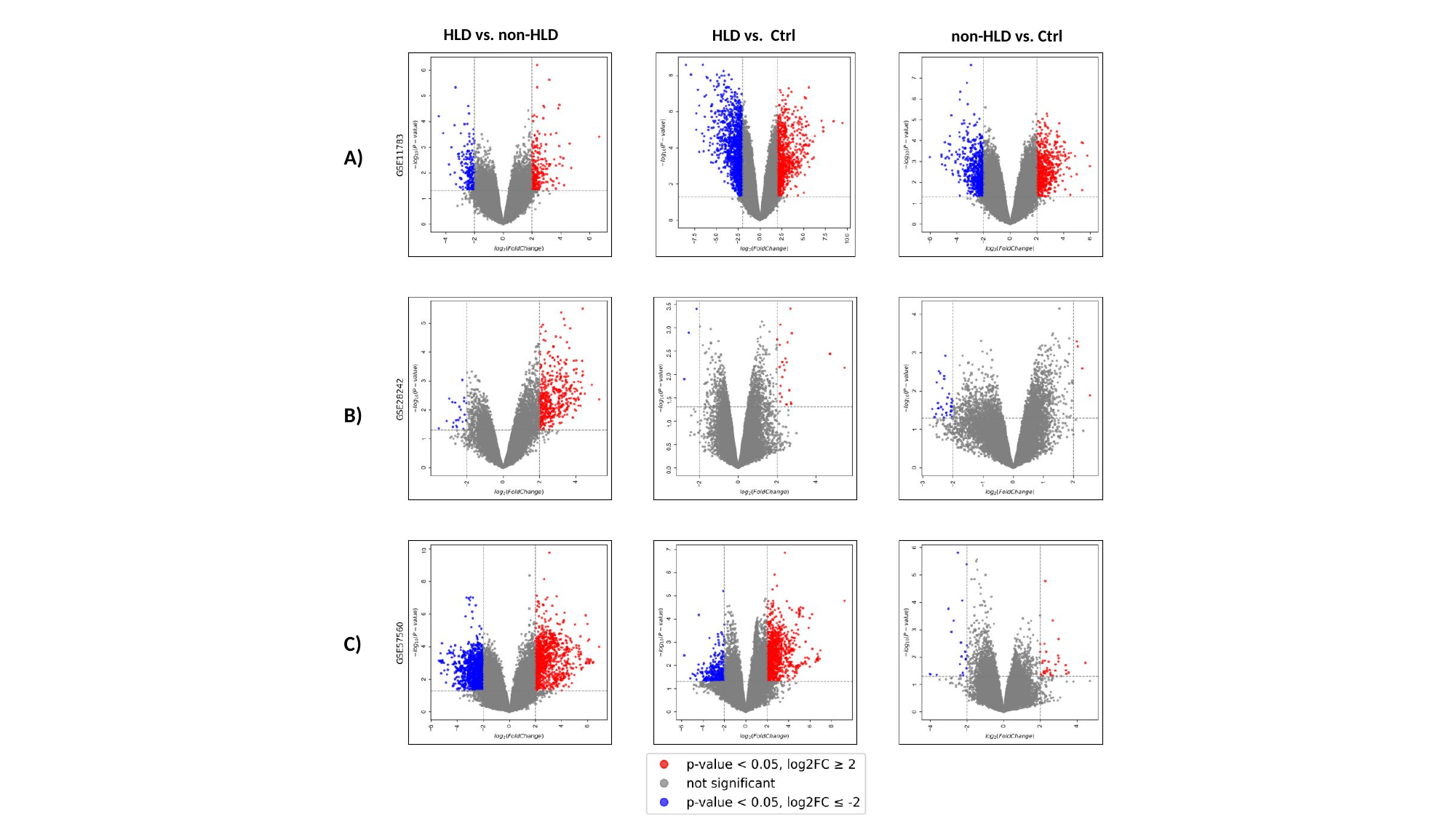

HLD vs. non-HLD
HLD vs. Ctrl
non-HLD vs. Ctrl
A)
B)
C)

## Slide 2
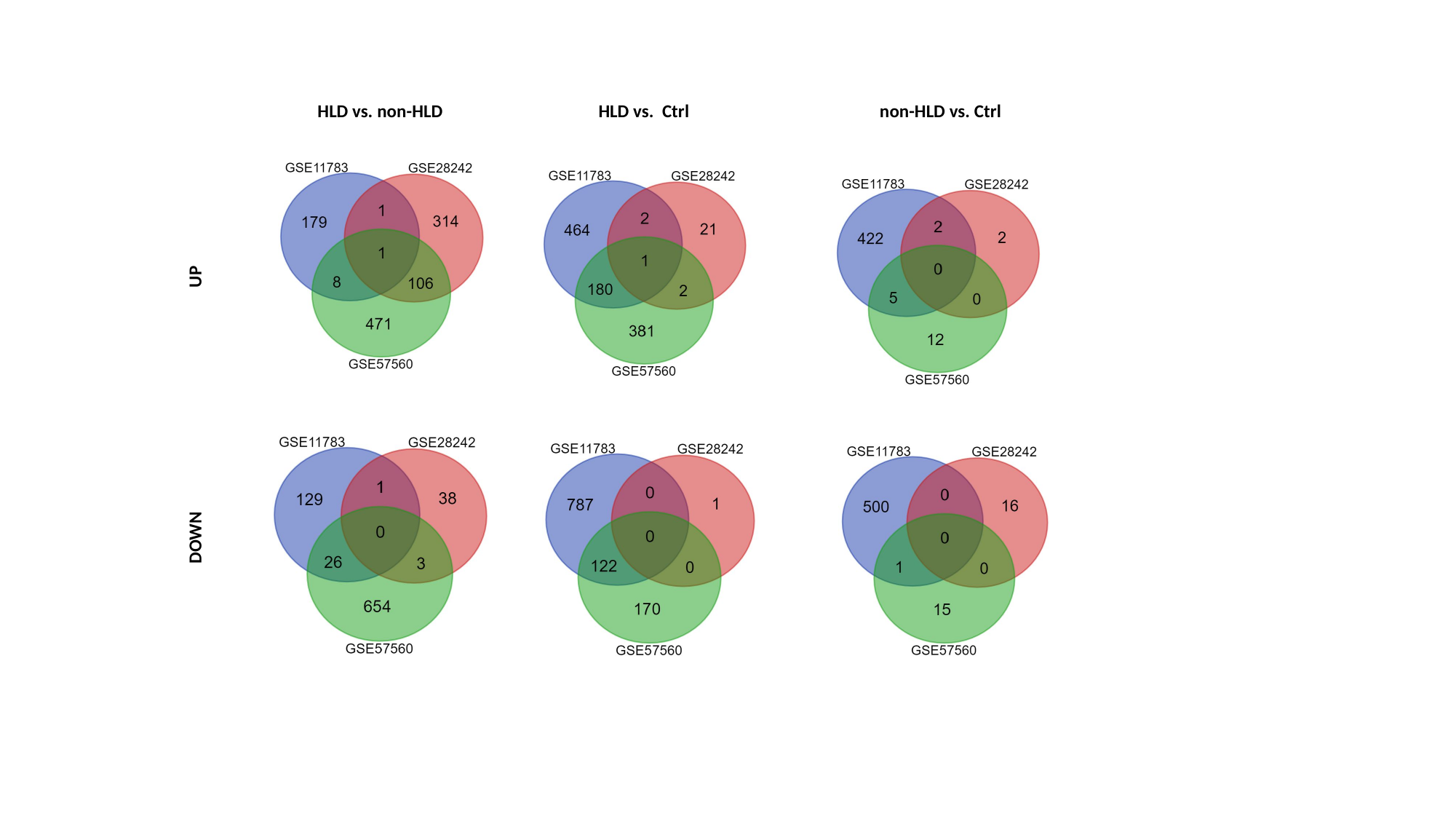

HLD vs. non-HLD
HLD vs. Ctrl
non-HLD vs. Ctrl
UP
DOWN

## Slide 3
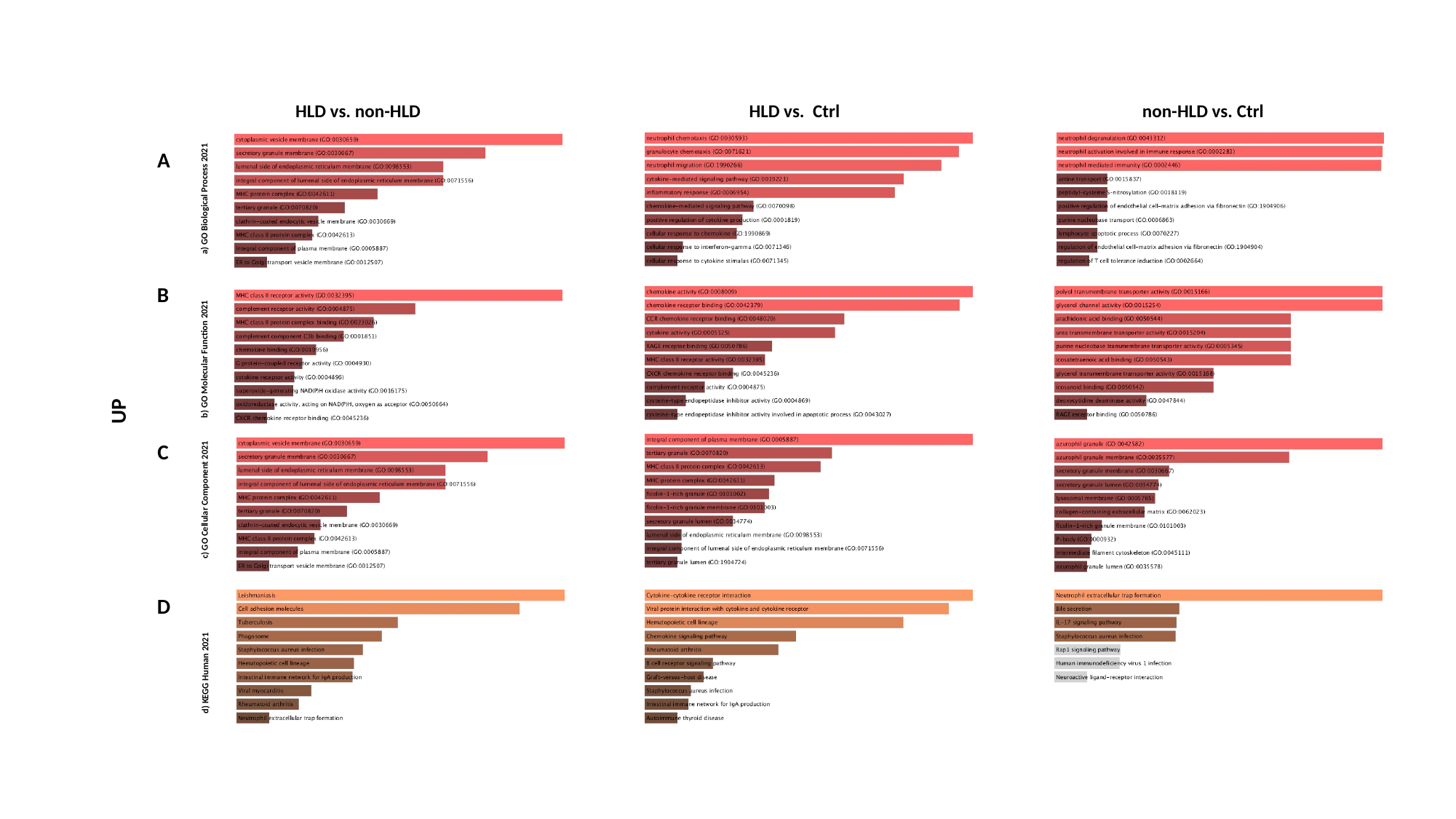

HLD vs. non-HLD
non-HLD vs. Ctrl
HLD vs. Ctrl
a) GO Biological Process 2021
b) GO Molecular Function 2021
UP
c) GO Cellular Component 2021
d) KEGG Human 2021
A
B
C
D

## Slide 4
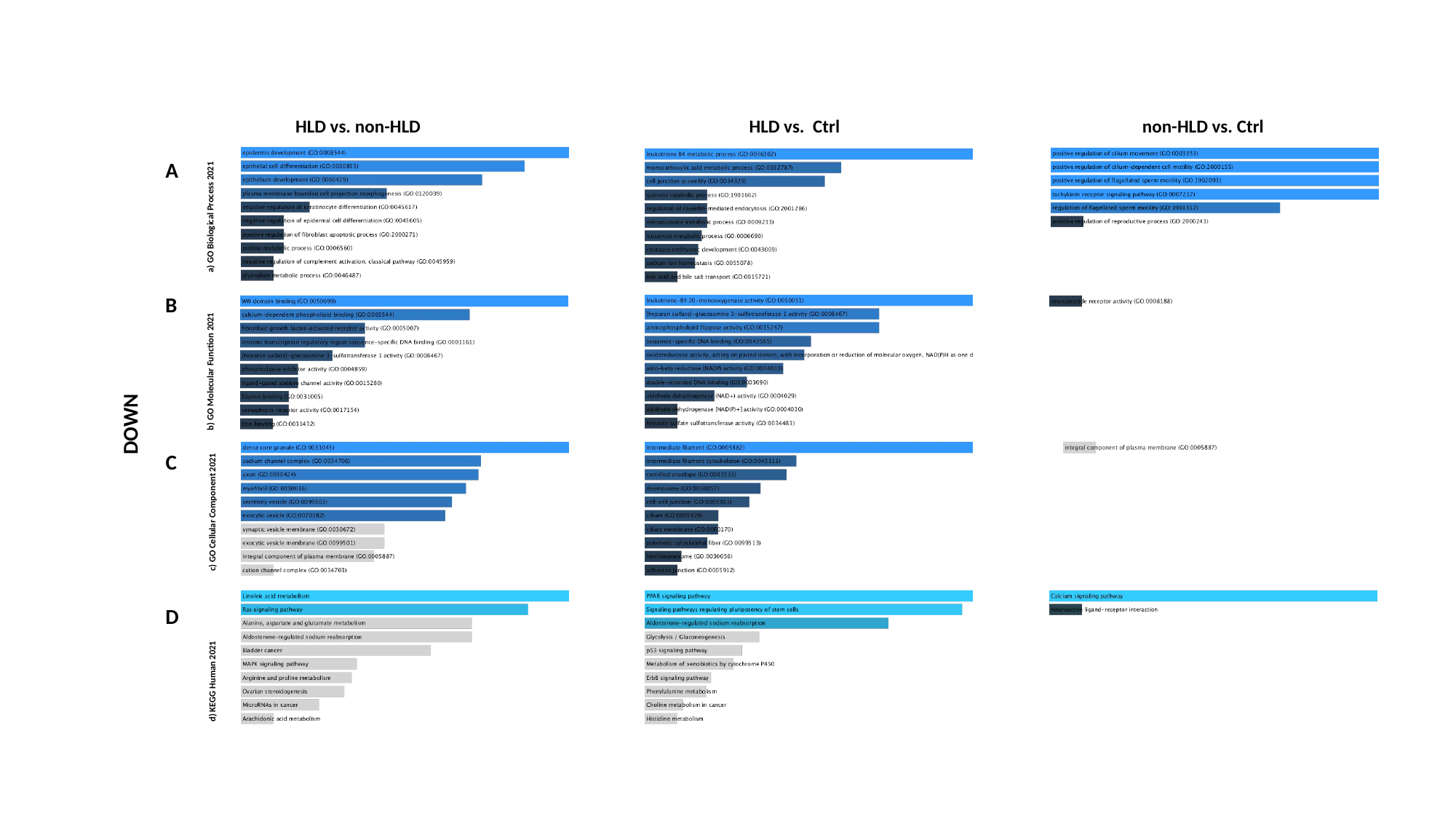

HLD vs. non-HLD
non-HLD vs. Ctrl
HLD vs. Ctrl
a) GO Biological Process 2021
b) GO Molecular Function 2021
DOWN
c) GO Cellular Component 2021
d) KEGG Human 2021
A
B
C
D

## Slide 5
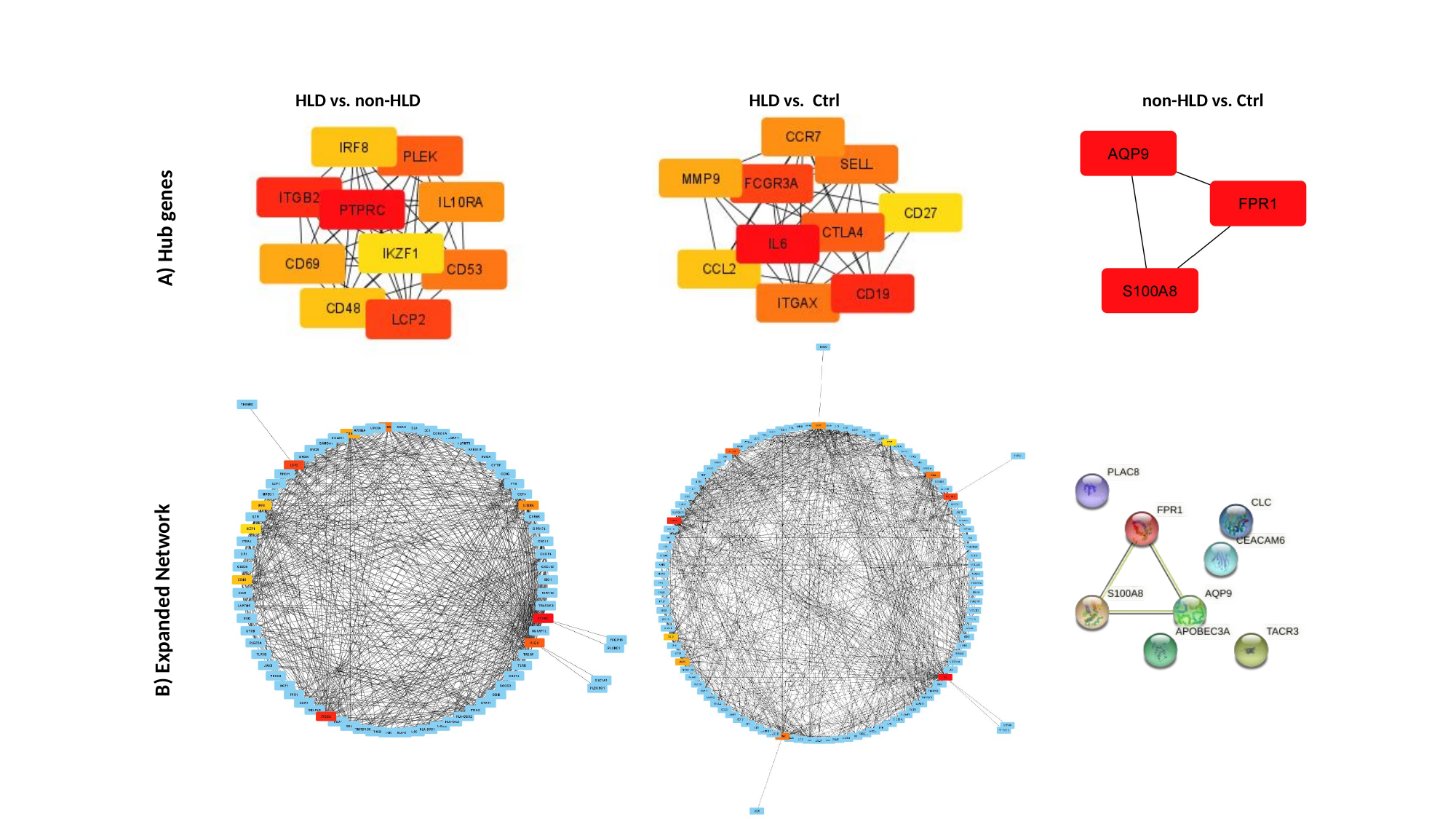

A) Hub genes
B) Expanded Network
HLD vs. non-HLD
non-HLD vs. Ctrl
HLD vs. Ctrl

## Slide 6
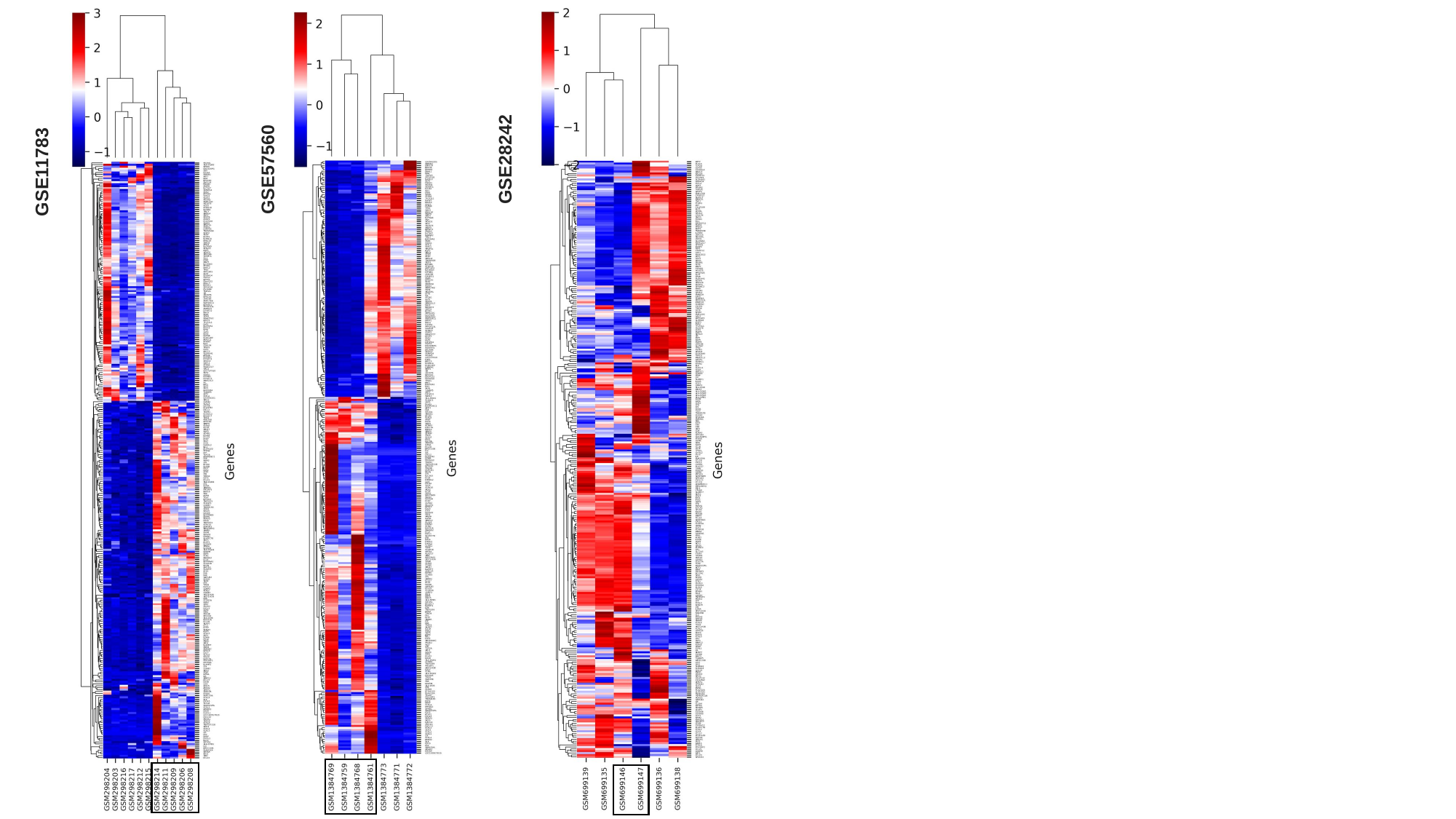

HLD vs. Ctrl
GSE11783
GSE28242
GSE57560

## Slide 7
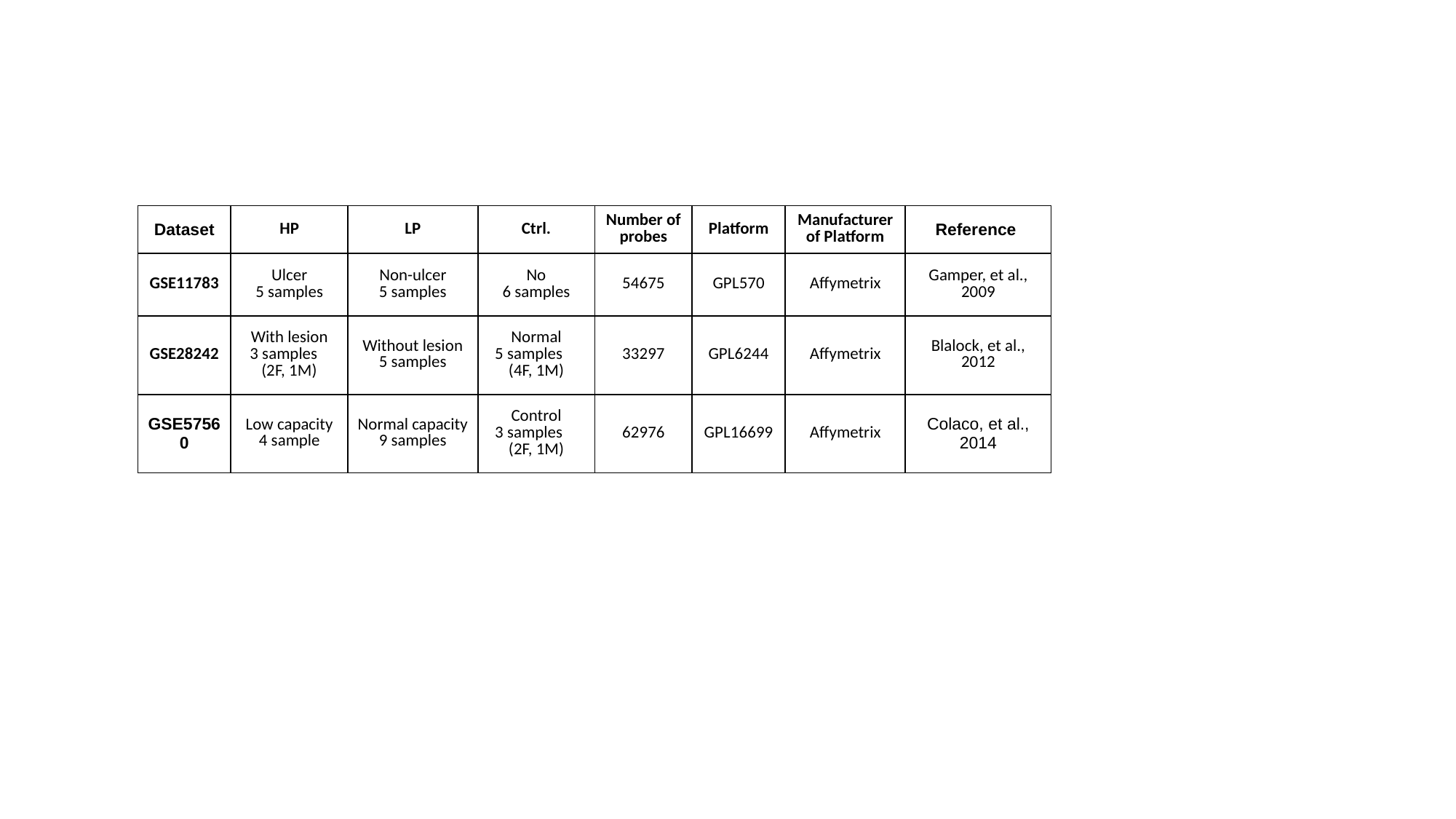

| Dataset | HP | LP | Ctrl. | Number of probes | Platform | Manufacturer of Platform | Reference |
| --- | --- | --- | --- | --- | --- | --- | --- |
| GSE11783 | Ulcer 5 samples | Non-ulcer 5 samples | No 6 samples | 54675 | GPL570 | Affymetrix | Gamper, et al., 2009 |
| GSE28242 | With lesion 3 samples (2F, 1M) | Without lesion 5 samples | Normal 5 samples (4F, 1M) | 33297 | GPL6244 | Affymetrix | Blalock, et al., 2012 |
| GSE57560 | Low capacity 4 sample | Normal capacity 9 samples | Control 3 samples (2F, 1M) | 62976 | GPL16699 | Affymetrix | Colaco, et al., 2014 |

## Slide 8
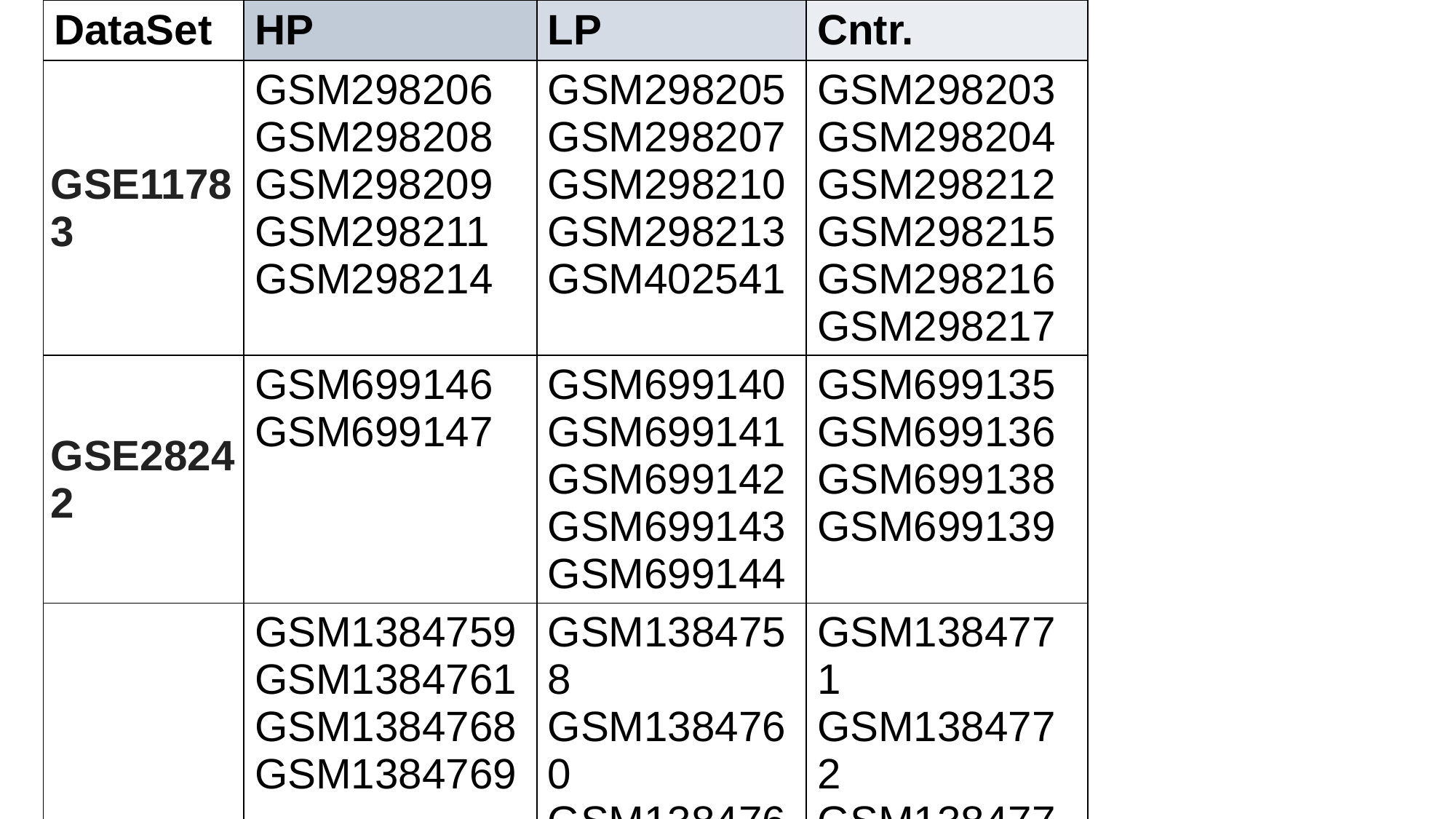

| DataSet | HP | LP | Cntr. |
| --- | --- | --- | --- |
| GSE11783 | GSM298206 GSM298208 GSM298209 GSM298211 GSM298214 | GSM298205 GSM298207 GSM298210 GSM298213 GSM402541 | GSM298203 GSM298204 GSM298212 GSM298215 GSM298216 GSM298217 |
| GSE28242 | GSM699146 GSM699147 | GSM699140 GSM699141 GSM699142 GSM699143 GSM699144 | GSM699135 GSM699136 GSM699138 GSM699139 |
| GSE57560 | GSM1384759 GSM1384761 GSM1384768 GSM1384769 | GSM1384758 GSM1384760 GSM1384762 GSM1384763 GSM1384764 GSM1384765 GSM1384766 GSM1384767 GSM1384770 | GSM1384771 GSM1384772 GSM1384773 |

## Slide 9
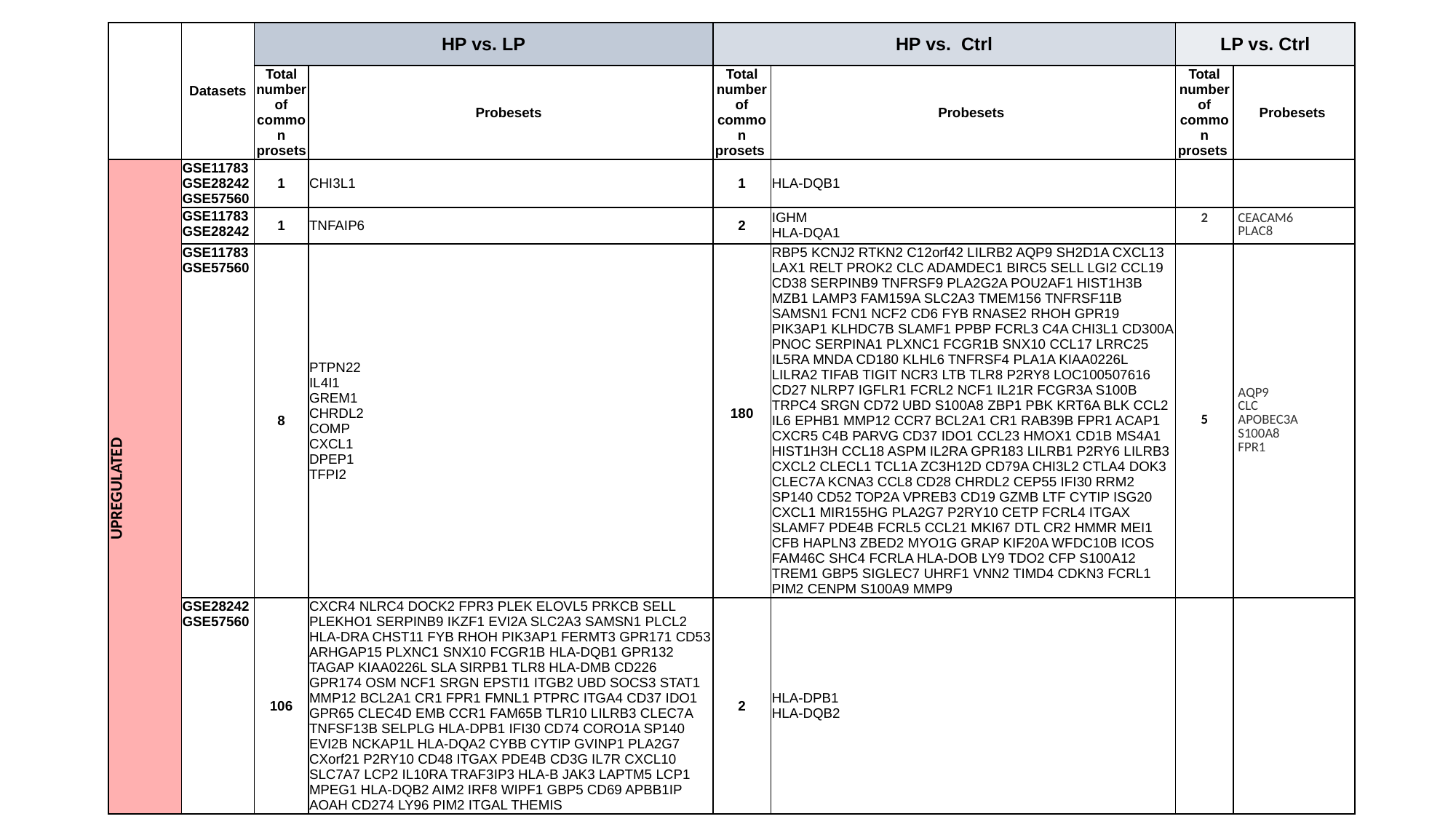

| | Datasets | HP vs. LP | HP vs. LP | HP vs. Ctrl | HP vs. Ctrl | LP vs. Ctrl | LP vs. Ctrl |
| --- | --- | --- | --- | --- | --- | --- | --- |
| | Datasets | Total number of common prosets | Probesets | Total number of common prosets | Probesets | Total number of common prosets | Probesets |
| UPREGULATED | GSE11783 GSE28242 GSE57560 | 1 | CHI3L1 | 1 | HLA-DQB1 | | |
| | GSE11783 GSE28242 | 1 | TNFAIP6 | 2 | IGHM HLA-DQA1 | 2 | CEACAM6 PLAC8 |
| | GSE11783 GSE57560 | 8 | PTPN22 IL4I1 GREM1 CHRDL2 COMP CXCL1 DPEP1 TFPI2 | 180 | RBP5 KCNJ2 RTKN2 C12orf42 LILRB2 AQP9 SH2D1A CXCL13 LAX1 RELT PROK2 CLC ADAMDEC1 BIRC5 SELL LGI2 CCL19 CD38 SERPINB9 TNFRSF9 PLA2G2A POU2AF1 HIST1H3B MZB1 LAMP3 FAM159A SLC2A3 TMEM156 TNFRSF11B SAMSN1 FCN1 NCF2 CD6 FYB RNASE2 RHOH GPR19 PIK3AP1 KLHDC7B SLAMF1 PPBP FCRL3 C4A CHI3L1 CD300A PNOC SERPINA1 PLXNC1 FCGR1B SNX10 CCL17 LRRC25 IL5RA MNDA CD180 KLHL6 TNFRSF4 PLA1A KIAA0226L LILRA2 TIFAB TIGIT NCR3 LTB TLR8 P2RY8 LOC100507616 CD27 NLRP7 IGFLR1 FCRL2 NCF1 IL21R FCGR3A S100B TRPC4 SRGN CD72 UBD S100A8 ZBP1 PBK KRT6A BLK CCL2 IL6 EPHB1 MMP12 CCR7 BCL2A1 CR1 RAB39B FPR1 ACAP1 CXCR5 C4B PARVG CD37 IDO1 CCL23 HMOX1 CD1B MS4A1 HIST1H3H CCL18 ASPM IL2RA GPR183 LILRB1 P2RY6 LILRB3 CXCL2 CLECL1 TCL1A ZC3H12D CD79A CHI3L2 CTLA4 DOK3 CLEC7A KCNA3 CCL8 CD28 CHRDL2 CEP55 IFI30 RRM2 SP140 CD52 TOP2A VPREB3 CD19 GZMB LTF CYTIP ISG20 CXCL1 MIR155HG PLA2G7 P2RY10 CETP FCRL4 ITGAX SLAMF7 PDE4B FCRL5 CCL21 MKI67 DTL CR2 HMMR MEI1 CFB HAPLN3 ZBED2 MYO1G GRAP KIF20A WFDC10B ICOS FAM46C SHC4 FCRLA HLA-DOB LY9 TDO2 CFP S100A12 TREM1 GBP5 SIGLEC7 UHRF1 VNN2 TIMD4 CDKN3 FCRL1 PIM2 CENPM S100A9 MMP9 | 5 | AQP9 CLC APOBEC3A S100A8 FPR1 |
| | GSE28242 GSE57560 | 106 | CXCR4 NLRC4 DOCK2 FPR3 PLEK ELOVL5 PRKCB SELL PLEKHO1 SERPINB9 IKZF1 EVI2A SLC2A3 SAMSN1 PLCL2 HLA-DRA CHST11 FYB RHOH PIK3AP1 FERMT3 GPR171 CD53 ARHGAP15 PLXNC1 SNX10 FCGR1B HLA-DQB1 GPR132 TAGAP KIAA0226L SLA SIRPB1 TLR8 HLA-DMB CD226 GPR174 OSM NCF1 SRGN EPSTI1 ITGB2 UBD SOCS3 STAT1 MMP12 BCL2A1 CR1 FPR1 FMNL1 PTPRC ITGA4 CD37 IDO1 GPR65 CLEC4D EMB CCR1 FAM65B TLR10 LILRB3 CLEC7A TNFSF13B SELPLG HLA-DPB1 IFI30 CD74 CORO1A SP140 EVI2B NCKAP1L HLA-DQA2 CYBB CYTIP GVINP1 PLA2G7 CXorf21 P2RY10 CD48 ITGAX PDE4B CD3G IL7R CXCL10 SLC7A7 LCP2 IL10RA TRAF3IP3 HLA-B JAK3 LAPTM5 LCP1 MPEG1 HLA-DQB2 AIM2 IRF8 WIPF1 GBP5 CD69 APBB1IP AOAH CD274 LY96 PIM2 ITGAL THEMIS | 2 | HLA-DPB1 HLA-DQB2 | | |

## Slide 10
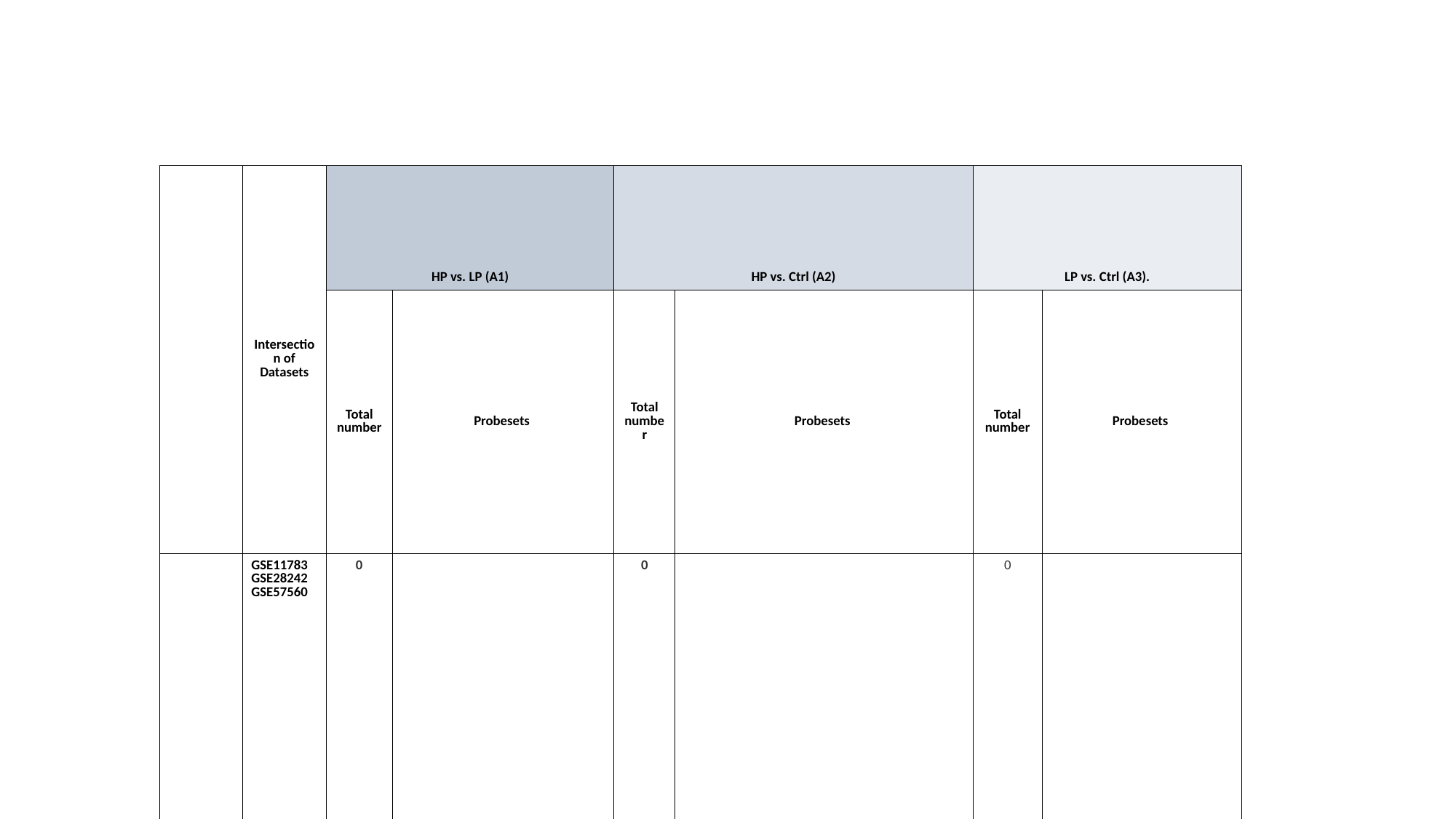

| | Intersection of Datasets | HP vs. LP (A1) | | HP vs. Ctrl (A2) | | LP vs. Ctrl (A3). | |
| --- | --- | --- | --- | --- | --- | --- | --- |
| | | Total number | Probesets | Total number | Probesets | Total number | Probesets |
| DOWNREGULATED | GSE11783 GSE28242 GSE57560 | 0 | | 0 | | 0 | |
| | GSE11783 GSE28242 | 1 | MYBPC1 | 0 | | 0 | |
| | GSE11783 GSE57560 | 26 | CPA6 UPK1A RIPK4 ALDH4A1 KLF5 PLXNB1 FMO5 FGFR3 SYT8 RAPGEFL1 NEBL SUSD4 SNX31 GRHL2 C10orf99 MUC15 ANXA9 C4orf19 NTF4 ELF5 HS3ST6 OVOL1 C1orf210 MFAP3L CYP2J2 TP63 | 122 | C1orf106 PTPRR REEP6 PKP1 GDA GPR143 CAPS IGSF3 PROM2 KIAA1522 MAB21L3 BMP3 SPAG16 ISL1 TMEM30B F2RL1 TFCP2L1 FABP5 PAK6 ALDH4A1 ATOH8 FAM110C ZSCAN4 TUBBP5 FAT2 FGFR3 PPARG RAPGEFL1 CNGA1 GRHL2 PERP SPINK5 FAM83H FSTL4 SERINC2 NIPAL4 PPP1R1B BTBD16 KIAA1217 FRMPD4 SPOCK3 GRHL1 NEDD4L SIX2 CDC42BPG S100A14 ABCC3 MAP7 HR HS3ST6 TPRXL LOC643201 LGALS4 IL20RA PWRN1 ESRP2 NRG2 CYP4F12 WIF1 GRTP1 HS3ST5 MFAP3L LRRC8E SOX15 DCDC2 HOXD1 SLITRK6 PKP2 CHMP4C CPA6 CAPN8 HAS3 PPP1R13L CHP2 CLCA4 SLC44A3 SLC5A7 CYP4F22 PLEK2 USP31 KRTCAP3 SH3YL1 FZD5 AQP3 FOXA1 ALDH3A1 PTGR1 AZGP1 TFAP2C KLF5 LAD1 PLEKHH1 SYT8 SNCG FBP1 CCDC169 PROM1 DST KRT7 FABP6 SHANK2 ACOXL C6orf132 AKR1C1 FERMT1 ELF5 SCNN1A SSH3 KSR2 DDR1 LOC727916 CYP4F11 KCNJ15 RPRM IVL ZNF214 DAPL1 TMC7 ATP8B1 TP63 CYP4B1 FAM174B | 1 | TACR3 |
| | GSE28242 GSE57560 | 3 | C2orf54 TMPRSS11E SCNN1B | 0 | | 0 | |

## Slide 11
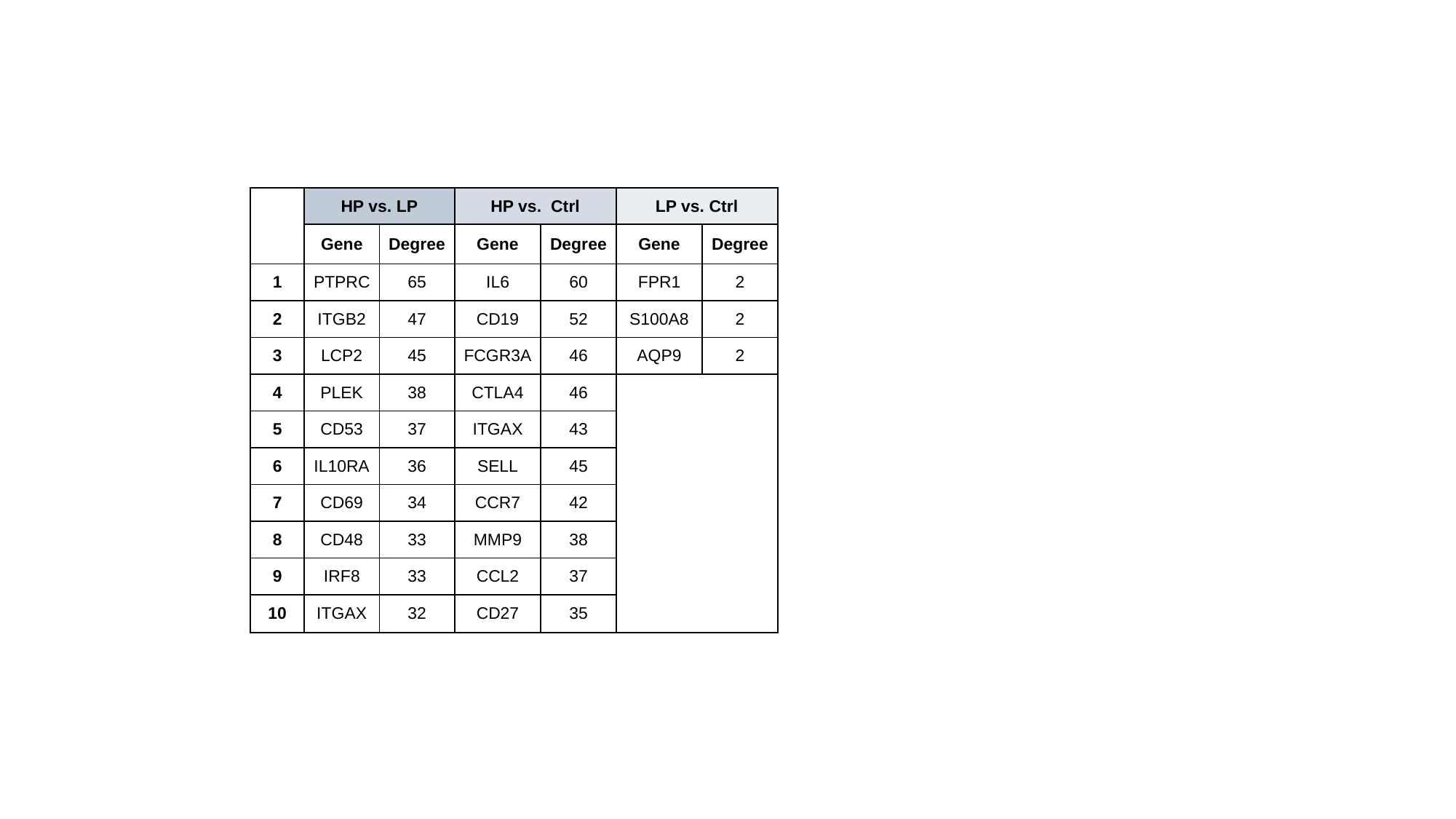

| | HP vs. LP | HP vs. LP | HP vs. Ctrl | HP vs. Ctrl | LP vs. Ctrl | LP vs. Ctrl |
| --- | --- | --- | --- | --- | --- | --- |
| | Gene | Degree | Gene | Degree | Gene | Degree |
| 1 | PTPRC | 65 | IL6 | 60 | FPR1 | 2 |
| 2 | ITGB2 | 47 | CD19 | 52 | S100A8 | 2 |
| 3 | LCP2 | 45 | FCGR3A | 46 | AQP9 | 2 |
| 4 | PLEK | 38 | CTLA4 | 46 | | |
| 5 | CD53 | 37 | ITGAX | 43 | | |
| 6 | IL10RA | 36 | SELL | 45 | | |
| 7 | CD69 | 34 | CCR7 | 42 | | |
| 8 | CD48 | 33 | MMP9 | 38 | | |
| 9 | IRF8 | 33 | CCL2 | 37 | | |
| 10 | ITGAX | 32 | CD27 | 35 | | |

## Slide 12
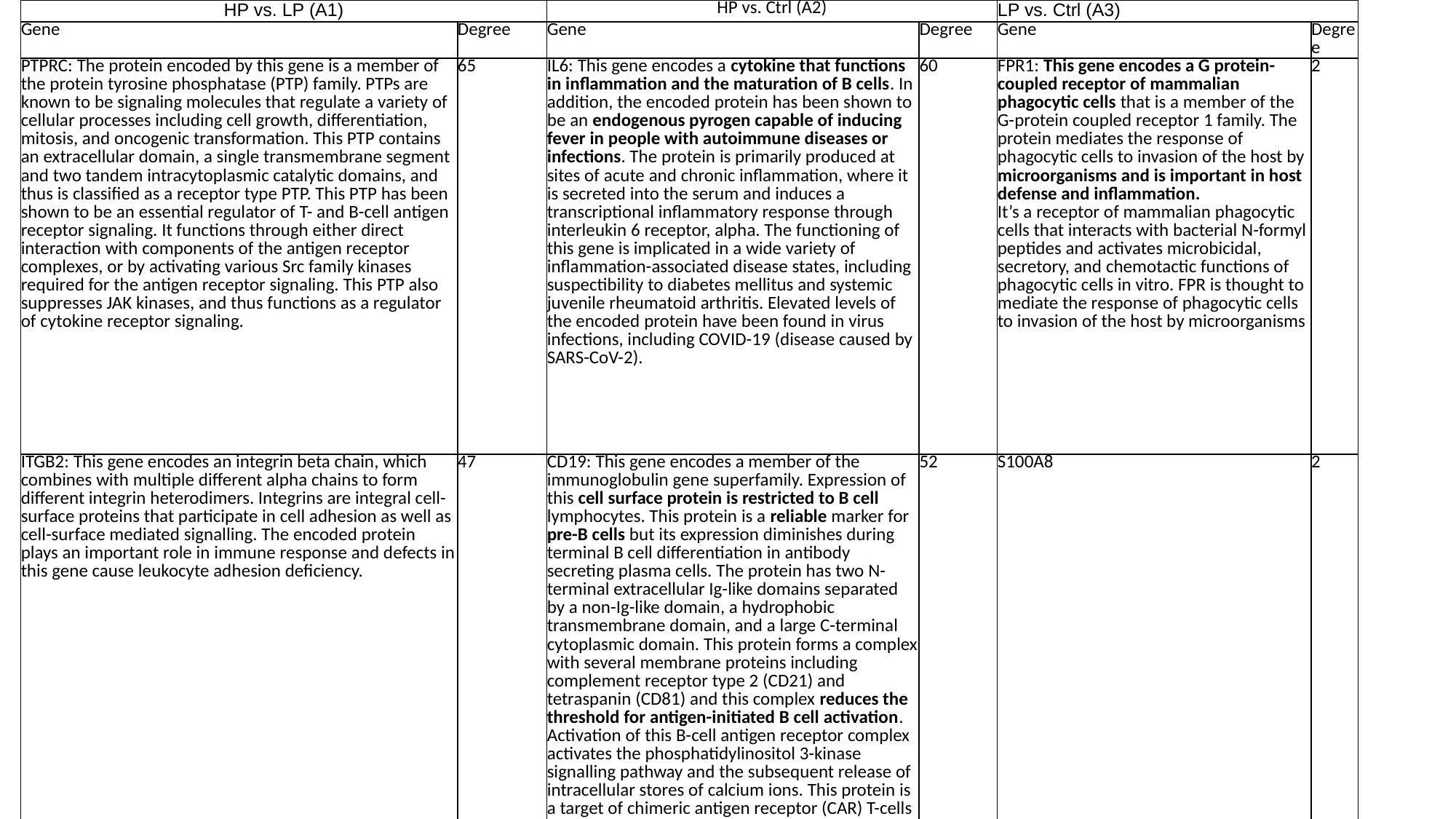

| HP vs. LP (A1) | | HP vs. Ctrl (A2) | | LP vs. Ctrl (A3) | |
| --- | --- | --- | --- | --- | --- |
| Gene | Degree | Gene | Degree | Gene | Degree |
| PTPRC: The protein encoded by this gene is a member of the protein tyrosine phosphatase (PTP) family. PTPs are known to be signaling molecules that regulate a variety of cellular processes including cell growth, differentiation, mitosis, and oncogenic transformation. This PTP contains an extracellular domain, a single transmembrane segment and two tandem intracytoplasmic catalytic domains, and thus is classified as a receptor type PTP. This PTP has been shown to be an essential regulator of T- and B-cell antigen receptor signaling. It functions through either direct interaction with components of the antigen receptor complexes, or by activating various Src family kinases required for the antigen receptor signaling. This PTP also suppresses JAK kinases, and thus functions as a regulator of cytokine receptor signaling. | 65 | IL6: This gene encodes a cytokine that functions in inflammation and the maturation of B cells. In addition, the encoded protein has been shown to be an endogenous pyrogen capable of inducing fever in people with autoimmune diseases or infections. The protein is primarily produced at sites of acute and chronic inflammation, where it is secreted into the serum and induces a transcriptional inflammatory response through interleukin 6 receptor, alpha. The functioning of this gene is implicated in a wide variety of inflammation-associated disease states, including suspectibility to diabetes mellitus and systemic juvenile rheumatoid arthritis. Elevated levels of the encoded protein have been found in virus infections, including COVID-19 (disease caused by SARS-CoV-2). | 60 | FPR1: This gene encodes a G protein-coupled receptor of mammalian phagocytic cells that is a member of the G-protein coupled receptor 1 family. The protein mediates the response of phagocytic cells to invasion of the host by microorganisms and is important in host defense and inflammation. It’s a receptor of mammalian phagocytic cells that interacts with bacterial N-formyl peptides and activates microbicidal, secretory, and chemotactic functions of phagocytic cells in vitro. FPR is thought to mediate the response of phagocytic cells to invasion of the host by microorganisms | 2 |
| ITGB2: This gene encodes an integrin beta chain, which combines with multiple different alpha chains to form different integrin heterodimers. Integrins are integral cell-surface proteins that participate in cell adhesion as well as cell-surface mediated signalling. The encoded protein plays an important role in immune response and defects in this gene cause leukocyte adhesion deficiency. | 47 | CD19: This gene encodes a member of the immunoglobulin gene superfamily. Expression of this cell surface protein is restricted to B cell lymphocytes. This protein is a reliable marker for pre-B cells but its expression diminishes during terminal B cell differentiation in antibody secreting plasma cells. The protein has two N-terminal extracellular Ig-like domains separated by a non-Ig-like domain, a hydrophobic transmembrane domain, and a large C-terminal cytoplasmic domain. This protein forms a complex with several membrane proteins including complement receptor type 2 (CD21) and tetraspanin (CD81) and this complex reduces the threshold for antigen-initiated B cell activation. Activation of this B-cell antigen receptor complex activates the phosphatidylinositol 3-kinase signalling pathway and the subsequent release of intracellular stores of calcium ions. This protein is a target of chimeric antigen receptor (CAR) T-cells used in the treatment of lymphoblastic leukemia. Mutations in this gene are associated with the disease common variable immunodeficiency 3 (CVID3) which results in a failure of B-cell differentiation and impaired secretion of immunoglobulins. CVID3 is characterized by hypogammaglobulinemia, an inability to mount an antibody response to antigen, and recurrent bacterial infections. Alternative splicing results in multiple transcript variants encoding distinct isoforms. | 52 | S100A8 | 2 |
| LCP2: This gene encodes an adapter protein that acts as a substrate of the T cell antigen receptor (TCR)-activated protein tyrosine kinase pathway. The encoded protein associates with growth factor receptor bound protein 2, and is thought to play a role TCR-mediated intracellular signal transduction. A similar protein in mouse plays a role in normal T-cell development and activation. Mice lacking this gene show subcutaneous and intraperitoneal fetal hemorrhaging, dysfunctional platelets and impaired viability. | 45 | FCGR3A: This gene encodes a receptor for the Fc portion of immunoglobulin G, and it is involved in the removal of antigen-antibody complexes from the circulation, as well as other responses, including antibody dependent cellular mediated cytotoxicity and antibody dependent enhancement of virus infections. This gene (FCGR3A) is highly similar to another nearby gene (FCGR3B) located on chromosome 1. The receptor encoded by this gene is expressed on natural killer (NK) cells as an integral membrane glycoprotein anchored through a transmembrane peptide, whereas FCGR3B is expressed on polymorphonuclear neutrophils (PMN) where the receptor is anchored through a phosphatidylinositol (PI) linkage. Mutations in this gene are associated with immunodeficiency 20, and have been linked to susceptibility to recurrent viral infections, susceptibility to systemic lupus erythematosus, and alloimmune neonatal neutropenia. Alternatively spliced transcript variants encoding different isoforms have been found for this gene | 46 | AQP9: The aquaporins are a family of water-selective membrane channels. This gene encodes a member of a subset of aquaporins called the aquaglyceroporins. This protein allows passage of a broad range of noncharged solutes and also stimulates urea transport and osmotic water permeability. This protein may also facilitate the uptake of glycerol in hepatic tissue . The encoded protein may also play a role in specialized leukocyte functions such as immunological response and bactericidal activity. Alternate splicing results in multiple transcript variants. | 2 |
| PLEK | 38 | CTLA4: This gene is a member of the immunoglobulin superfamily and encodes a protein which transmits an inhibitory signal to T cells. The protein contains a V domain, a transmembrane domain, and a cytoplasmic tail. Alternate transcriptional splice variants, encoding different isoforms, have been characterized. The membrane-bound isoform functions as a homodimer interconnected by a disulfide bond, while the soluble isoform functions as a monomer. Mutations in this gene have been associated with insulin-dependent diabetes mellitus, Graves disease, Hashimoto thyroiditis, celiac disease, systemic lupus erythematosus, thyroid-associated orbitopathy, and other autoimmune diseases. | 46 | | |
| CD53: The protein encoded by this gene is a member of the transmembrane 4 superfamily, also known as the tetraspanin family. Most of these members are cell-surface proteins that are characterized by the presence of four hydrophobic domains. The proteins mediate signal transduction events that play a role in the regulation of cell development, activation, growth and motility. This encoded protein is a cell surface glycoprotein that is known to complex with integrins. It contributes to the transduction of CD2-generated signals in T cells and natural killer cells and has been suggested to play a role in growth regulation. Familial deficiency of this gene has been linked to an immunodeficiency associated with recurrent infectious diseases caused by bacteria, fungi and viruses. | 37 | ITGAX: This gene encodes the integrin alpha X chain protein. Integrins are heterodimeric integral membrane proteins composed of an alpha chain and a beta chain. This protein combines with the beta 2 chain (ITGB2) to form a leukocyte-specific integrin referred to as inactivated-C3b (iC3b) receptor 4 (CR4). The alpha X beta 2 complex seems to overlap the properties of the alpha M beta 2 integrin in the adherence of neutrophils and monocytes to stimulated endothelium cells, and in the phagocytosis of complement coated particles. Two transcript variants encoding different isoforms have been found for this gene. | 43 | | |
| IL10RA: The protein encoded by this gene is a receptor for interleukin 10. This protein is structurally related to interferon receptors. It has been shown to mediate the immunosuppressive signal of interleukin 10, and thus inhibits the synthesis of proinflammatory cytokines. This receptor is reported to promote survival of progenitor myeloid cells through the insulin receptor substrate-2/PI 3-kinase/AKT pathway. Activation of this receptor leads to tyrosine phosphorylation of JAK1 and TYK2 kinases | 36 | SELL: This gene encodes a cell surface adhesion molecule that belongs to a family of adhesion/homing receptors. The encoded protein contains a C-type lectin-like domain, a calcium-binding epidermal growth factor-like domain, and two short complement-like repeats. The gene product is required for binding and subsequent rolling of leucocytes on endothelial cells, facilitating their migration into secondary lymphoid organs and inflammation sites. Single-nucleotide polymorphisms in this gene have been associated with various diseases including immunoglobulin A nephropathy. Alternatively spliced transcript variants have been found for this gene. | 45 | | |
| CD69: This gene encodes a member of the calcium dependent lectin superfamily of type II transmembrane receptors. Expression of the encoded protein is induced upon activation of T lymphocytes, and may play a role in proliferation. Furthermore, the protein may act to transmit signals in natural killer cells and platelets. | 34 | CCR7: The protein encoded by this gene is a member of the G protein-coupled receptor family. This receptor was identified as a gene induced by the Epstein-Barr virus (EBV), and is thought to be a mediator of EBV effects on B lymphocytes. This receptor is expressed in various lymphoid tissues and activates B and T lymphocytes. It has been shown to control the migration of memory T cells to inflamed tissues, as well as stimulate dendritic cell maturation. The chemokine (C-C motif) ligand 19 (CCL19/ECL) has been reported to be a specific ligand of this receptor. Signals mediated by this receptor regulate T cell homeostasis in lymph nodes, and may also function in the activation and polarization of T cells, and in chronic inflammation pathogenesis. | 42 | | |
| CD48: This gene encodes a member of the CD2 subfamily of immunoglobulin-like receptors which includes SLAM (signaling lymphocyte activation molecules) proteins. The encoded protein is found on the surface of lymphocytes and other immune cells, dendritic cells and endothelial cells, and participates in activation and differentiation pathways in these cells. The encoded protein does not have a transmembrane domain, however, but is held at the cell surface by a GPI anchor via a C-terminal domain which maybe cleaved to yield a soluble form of the receptor. | 33 | MMP9: Proteins of the matrix metalloproteinase (MMP) family are involved in the breakdown of extracellular matrix in normal physiological processes, such as embryonic development, reproduction, and tissue remodeling, as well as in disease processes, such as arthritis and metastasis. Most MMP's are secreted as inactive proproteins which are activated when cleaved by extracellular proteinases. The enzyme encoded by this gene degrades type IV and V collagens. Studies in rhesus monkeys suggest that the enzyme is involved in IL-8-induced mobilization of hematopoietic progenitor cells from bone marrow, and murine studies suggest a role in tumor-associated tissue remodeling. | 38 | | |
| IRF8: Interferon consensus sequence-binding protein (ICSBP) is a transcription factor of the interferon (IFN) regulatory factor (IRF) family. Proteins of this family are composed of a conserved DNA-binding domain in the N-terminal region and a divergent C-terminal region that serves as the regulatory domain. The IRF family proteins bind to the IFN-stimulated response element (ISRE) and regulate expression of genes stimulated by type I IFNs, namely IFN-alpha and IFN-beta. IRF family proteins also control expression of IFN-alpha and IFN-beta-regulated genes that are induced by viral infection. | 33 | CCL2: This gene is one of several cytokine genes clustered on the q-arm of chromosome 17. Chemokines are a superfamily of secreted proteins involved in immunoregulatory and inflammatory processes. The superfamily is divided into four subfamilies based on the arrangement of N-terminal cysteine residues of the mature peptide. This chemokine is a member of the CC subfamily which is characterized by two adjacent cysteine residues. This cytokine displays chemotactic activity for monocytes and basophils but not for neutrophils or eosinophils. It has been implicated in the pathogenesis of diseases characterized by monocytic infiltrates, like psoriasis, rheumatoid arthritis and atherosclerosis. It binds to chemokine receptors CCR2 and CCR4. Elevated expression of the encoded protein is associated with severe acute respiratory syndrome coronavirus 2 | 37 | | |
| ITGAX: This gene encodes the integrin alpha X chain protein. Integrins are heterodimeric integral membrane proteins composed of an alpha chain and a beta chain. This protein combines with the beta 2 chain (ITGB2) to form a leukocyte-specific integrin referred to as inactivated-C3b (iC3b) receptor 4 (CR4). The alpha X beta 2 complex seems to overlap the properties of the alpha M beta 2 integrin in the adherence of neutrophils and monocytes to stimulated endothelium cells, and in the phagocytosis of complement coated particles | 32 | CD27: The protein encoded by this gene is a member of the TNF-receptor superfamily. This receptor is required for generation and long-term maintenance of T cell immunity. It binds to ligand CD70, and plays a key role in regulating B-cell activation and immunoglobulin synthesis. This receptor transduces signals that lead to the activation of NF-kappaB and MAPK8/JNK. Adaptor proteins TRAF2 and TRAF5 have been shown to mediate the signaling process of this receptor. CD27-binding protein (SIVA), a proapoptotic protein, can bind to this receptor and is thought to play an important role in the apoptosis induced by this receptor. | 35 | | |
