# Supplementary material for: Defining Molecular Treatment Targets for Bladder Pain Syndrome/Interstitial Cystitis: Uncovering Adhesion Molecules
Source: Front Pharmacol. 2022 Mar 25;13:780855. doi: 10.3389/fphar.2022.780855 (PMC8990855; doi:10.3389/fphar.2022.780855)
Supplement: Supplementary file 5 [file DataSheet1.docx]

**Table S-1:** Number of samples and probes, sex (when available) and platform information of each GEO dataset.

| **Dataset** | **HLD** | **Non-HLD** | **Ctrl.** | **Number of**  **probes** | **Platform** | **Manufacturer**  **of Platform** | **Reference** |
| --- | --- | --- | --- | --- | --- | --- | --- |
| **GSE11783** | Ulcer  5 samples | Non-ulcer  5 samples | Normal  6 samples | 54675 | GPL570 | Affymetrix | Gamper, et al., 2009 |
| **GSE28242** | With lesion  3 samples  (2F, 1M) | Without lesion  5 samples | Normal  5 samples  (4F, 1M) | 33297 | GPL6244 | Affymetrix | Blalock, et al., 2012 |
| **GSE57560** | Low capacity  4 sample | Normal capacity  9 samples | Control  3 samples  (2F, 1M) | 62976 | GPL16699 | Affymetrix | Colaco, et al., 2014 |

**Table S2:** Patient and control samples of each dataset as retrieved from the database.

| **DataSet** | **HLD** | **Non-HLD** | **Ctrl.** |
| --- | --- | --- | --- |
| **GSE11783** | GSM298206 GSM298208 GSM298209 GSM298211 GSM298214 | GSM298205 GSM298207 GSM298210 GSM298213 GSM402541 | GSM298203 GSM298204 GSM298212 GSM298215 GSM298216  GSM298217 |
| **GSE28242** | GSM699146 GSM699147 | GSM699140 GSM699141 GSM699142 GSM699143 GSM699144 | GSM699135 GSM699136 GSM699138 GSM699139 |
| **GSE57560** | GSM1384759 GSM1384761 GSM1384768 GSM1384769 | GSM1384758 GSM1384760 GSM1384762 GSM1384763 GSM1384764  GSM1384765 GSM1384766 GSM1384767  GSM1384770 | GSM1384771 GSM1384772 GSM1384773 |

**Table S3:** The total number and list of common probesets of up-regulated probesets in respect to Venn analysis.

| **UPREGULATED PROBES** | **Intersection of Datasets** | **HLD vs. non-HLD** | | **HLD vs. Ctrl** | | **non-HLD vs. Ctrl** | |
| --- | --- | --- | --- | --- | --- | --- | --- |
|  |  | **Total number of common prosets** | **Probesets** | **Total number of common prosets** | **Probesets** | **Total number of common prosets** | **Probesets** |
|  | **GSE11783**  **GSE28242**  **GSE57560** | **1** | CHI3L1 | **1** | HLA-DQB1 | **0** | - |
|  | **GSE11783**  **GSE28242** | **1** | TNFAIP6 | **2** | IGHM  HLA-DQA1 | **2** | CEACAM6  PLAC8 |
|  | **GSE11783**  **GSE57560** | **8** | PTPN22  IL4I1  GREM1  CHRDL2  COMP  CXCL1  DPEP1  TFPI2 | **180** | RBP5 KCNJ2 RTKN2 C12orf42 LILRB2 AQP9 SH2D1A CXCL13 LAX1 RELT PROK2 CLC ADAMDEC1 BIRC5 SELL LGI2 CCL19 CD38 SERPINB9 TNFRSF9 PLA2G2A POU2AF1 HIST1H3B MZB1 LAMP3 FAM159A SLC2A3 TMEM156 TNFRSF11B SAMSN1 FCN1 NCF2 CD6 FYB RNASE2 RHOH GPR19 PIK3AP1 KLHDC7B SLAMF1 PPBP FCRL3 C4A CHI3L1 CD300A PNOC SERPINA1 PLXNC1 FCGR1B SNX10 CCL17 LRRC25 IL5RA MNDA CD180 KLHL6 TNFRSF4 PLA1A KIAA0226L LILRA2 TIFAB TIGIT NCR3 LTB TLR8 P2RY8 LOC100507616 CD27 NLRP7 IGFLR1 FCRL2 NCF1 IL21R FCGR3A S100B TRPC4 SRGN CD72 UBD S100A8 ZBP1 PBK KRT6A BLK CCL2 IL6 EPHB1 MMP12 CCR7 BCL2A1 CR1 RAB39B FPR1 ACAP1 CXCR5 C4B PARVG CD37 IDO1 CCL23 HMOX1 CD1B MS4A1 HIST1H3H CCL18 ASPM IL2RA GPR183 LILRB1 P2RY6 LILRB3 CXCL2 CLECL1 TCL1A ZC3H12D CD79A CHI3L2 CTLA4 DOK3 CLEC7A KCNA3 CCL8 CD28 CHRDL2 CEP55 IFI30 RRM2 SP140 CD52 TOP2A VPREB3 CD19 GZMB LTF CYTIP ISG20 CXCL1 MIR155HG PLA2G7 P2RY10 CETP FCRL4 ITGAX SLAMF7 PDE4B FCRL5 CCL21 MKI67 DTL CR2 HMMR MEI1 CFB HAPLN3 ZBED2 MYO1G GRAP KIF20A WFDC10B ICOS FAM46C SHC4 FCRLA HLA-DOB LY9 TDO2 CFP S100A12 TREM1 GBP5 SIGLEC7 UHRF1 VNN2 TIMD4 CDKN3 FCRL1 PIM2 CENPM S100A9 MMP9 | **5** | AQP9  CLC  APOBEC3A  S100A8  FPR1 |
|  | **GSE28242**  **GSE57560** | **106** | CXCR4 NLRC4 DOCK2 FPR3 PLEK ELOVL5 PRKCB SELL PLEKHO1 SERPINB9 IKZF1 EVI2A SLC2A3 SAMSN1 PLCL2 HLA-DRA CHST11 FYB RHOH PIK3AP1 FERMT3 GPR171 CD53 ARHGAP15 PLXNC1 SNX10 FCGR1B HLA-DQB1 GPR132 TAGAP KIAA0226L SLA SIRPB1 TLR8 HLA-DMB CD226 GPR174 OSM NCF1 SRGN EPSTI1 ITGB2 UBD SOCS3 STAT1 MMP12 BCL2A1 CR1 FPR1 FMNL1 PTPRC ITGA4 CD37 IDO1 GPR65 CLEC4D EMB CCR1 FAM65B TLR10 LILRB3 CLEC7A TNFSF13B SELPLG HLA-DPB1 IFI30 CD74 CORO1A SP140 EVI2B NCKAP1L HLA-DQA2 CYBB CYTIP GVINP1 PLA2G7 CXorf21 P2RY10 CD48 ITGAX PDE4B CD3G IL7R CXCL10 SLC7A7 LCP2 IL10RA TRAF3IP3 HLA-B JAK3 LAPTM5 LCP1 MPEG1 HLA-DQB2 AIM2 IRF8 WIPF1 GBP5 CD69 APBB1IP AOAH CD274 LY96 PIM2 ITGAL THEMIS | **2** | HLA-DPB1  HLA-DQB2 | **0** | - |

**Table S-4:** The total number and list of common downregulated genes/proteins in respect to Venn analysis.

| **DOWNREGULATED** | **Intersection of Datasets** | **HLD vs. non-HLD** | | **HLD vs. Ctrl** | | **Non-HLD vs. Ctrl** | |
| --- | --- | --- | --- | --- | --- | --- | --- |
|  |  | **Total number of common prosets** | **Probesets** | **Total number of common prosets** | **Probesets** | **Total number of common prosets** | **Probesets** |
|  | **GSE11783  GSE28242  GSE57560** | **0** |  | **0** |  | **0** |  |
|  | **GSE11783  GSE28242** | **1** | MYBPC1 | **0** |  | **0** |  |
|  | **GSE11783  GSE57560** | **26** | CPA6 UPK1A RIPK4 ALDH4A1 KLF5 PLXNB1 FMO5 FGFR3 SYT8 RAPGEFL1 NEBL SUSD4 SNX31 GRHL2 C10orf99 MUC15 ANXA9 C4orf19 NTF4 ELF5 HS3ST6 OVOL1 C1orf210 MFAP3L CYP2J2 TP63 | **122** | C1orf106 PTPRR REEP6 PKP1 GDA GPR143 CAPS IGSF3 PROM2 KIAA1522 MAB21L3 BMP3 SPAG16 ISL1 TMEM30B F2RL1 TFCP2L1 FABP5 PAK6 ALDH4A1 ATOH8 FAM110C ZSCAN4 TUBBP5 FAT2 FGFR3 PPARG RAPGEFL1 CNGA1 GRHL2 PERP SPINK5 FAM83H FSTL4 SERINC2 NIPAL4 PPP1R1B BTBD16 KIAA1217 FRMPD4 SPOCK3 GRHL1 NEDD4L SIX2 CDC42BPG S100A14 ABCC3 MAP7 HR HS3ST6 TPRXL LOC643201 LGALS4 IL20RA PWRN1 ESRP2 NRG2 CYP4F12 WIF1 GRTP1 HS3ST5 MFAP3L LRRC8E SOX15 DCDC2 HOXD1 SLITRK6 PKP2 CHMP4C CPA6 CAPN8 HAS3 PPP1R13L CHP2 CLCA4 SLC44A3 SLC5A7 CYP4F22 PLEK2 USP31 KRTCAP3 SH3YL1 FZD5 AQP3 FOXA1 ALDH3A1 PTGR1 AZGP1 TFAP2C KLF5 LAD1 PLEKHH1 SYT8 SNCG FBP1 CCDC169 PROM1 DST KRT7 FABP6 SHANK2 ACOXL C6orf132 AKR1C1 FERMT1 ELF5 SCNN1A SSH3 KSR2 DDR1 LOC727916 CYP4F11 KCNJ15 RPRM IVL ZNF214 DAPL1 TMC7 ATP8B1 TP63 CYP4B1 FAM174B | **1** | TACR3 |
|  | **GSE28242  GSE57560** | **3** | C2orf54 TMPRSS11E SCNN1B | **0** |  | **0** |  |
